# Maintenance of *Xist* Imprinting Depends on Chromatin Condensation State and *Rnf12* Dosage in Mice

**DOI:** 10.1371/journal.pgen.1006375

**Published:** 2016-10-27

**Authors:** Atsushi Fukuda, Atsushi Mitani, Toshiyuki Miyashita, Takashi Sado, Akihiro Umezawa, Hidenori Akutsu

**Affiliations:** 1 Center for Regenerative Medicine, National Research Institute for Child Health and Development, Okura, Setagaya, Tokyo, Japan; 2 Department of Molecular Genetics, Kitasato University Graduate School of Medical Sciences, Kitasato, Minami, Sagamihara, Kanagawa, Japan; 3 Department of Advanced Bioscience, Graduate School of Agriculture, Kindai University, Nakamachi, Nara, Japan; 4 Department of Stem Cell Research, Fukushima Medical University, Hikarigaoka, Fukushima City, Fukushima, Japan; Stanford University School of Medicine, UNITED STATES

## Abstract

In female mammals, activation of *Xist* (X-inactive specific transcript) is essential for establishment of X chromosome inactivation. During early embryonic development in mice, paternal *Xist* is preferentially expressed whereas maternal *Xist* (Xm-*Xist*) is silenced. Unlike autosomal imprinted genes, *Xist* imprinting for Xm-*Xist* silencing was erased in cloned or parthenogenetic but not fertilized embryos. However, the molecular mechanism underlying the variable nature of Xm-*Xist* imprinting is poorly understood. Here, we revealed that Xm-*Xist* silencing depends on chromatin condensation states at the *Xist*/*Tsix* genomic region and on *Rnf12* expression levels. In early preimplantation, chromatin decondensation via H3K9me3 loss and histone acetylation gain caused Xm-*Xist* derepression irrespective of embryo type. Although the presence of the paternal genome during pronuclear formation impeded Xm-*Xist* derepression, Xm-*Xist* was robustly derepressed when the maternal genome was decondensed before fertilization. Once Xm-*Xist* was derepressed by chromatin alterations, the derepression was stably maintained and rescued XmXp^Δ^ lethality, indicating that loss of Xm-*Xist* imprinting was irreversible. In late preimplantation, Oct4 served as a chromatin opener to create transcriptional permissive states at Xm-*Xist*/*Tsix* genomic loci. In parthenogenetic embryos, *Rnf12* overdose caused Xm-*Xist* derepression via Xm-*Tsix* repression; physiological *Rnf12* levels were essential for Xm-*Xist* silencing maintenance in fertilized embryos. Thus, chromatin condensation and fine-tuning of *Rnf12* dosage were crucial for *Xist* imprint maintenance by silencing Xm-*Xist*.

## Author Summary

X-inactive specific transcript (*Xist*) is essential a large non-coding RNA for establishment of X chromosome inactivation in female mammals. The aberrant X chromosome inactivation critically affects cellular viability. Therefore, spatiotemporal regulation of *Xist* expression is required for proper development. In mice, *Xist* expression is imprinted in early embryonic development and maternal *Xist* is never expressed during preimplantation phases irrespective of the presence of Xist activator, maternal Rnf12. Generally, parental origin-specific expression pattern of autosomal imprinted genes is maintained in various types of embryos. However, *Xist* imprinting for transcriptional silencing of maternal *Xist* was erased in cloned or parthenogenetic but not fertilized embryos. Here, we dissect the molecular mechanism underlying the variable nature of *Xist* imprinting. We show that in fertilized embryos, chromatin condensation states are essential maternal *Xist* repression in early preimplantation phases, whereas at late preimplantation stages, pluripotency factor Oct4 serves as a chromatin opener and the maintenance of *Xist* silencing depends on *Rnf12* expression dosage. Although the Oct4 mediated chromatin decondensation also occurs in parthenogetic embryos, *Rnf12* overdose causes maternal *Xist* derepression at late preimplantation phases. Thus these findings reveal that the chromatin regulation by pluripotency factor and *Xist* activator dose define *Xist* imprinting state.

## Introduction

In mice, the expression of *Xist*, an essential non-coding RNA for the initiation of X-chromosome inactivation (XCI) [1–3], commences around early preimplantation phases [4,5]. The expression pattern during preimplantation phases is imprinted; paternal *Xist* (Xp-*Xist*) is activated and maternal *Xist* (Xm-*Xist*) is never expressed although the *Xist* activator, Rnf12/Rlim [6,7], is abundantly deposited in oocytes [8]. The predominant expression of Xp-*Xist* is reprogrammed in embryonic-tissues after implantation and maintained in extra-embryonic tissues [5,9]. Therefore, Xp-*Xist* mutation causes embryonic lethality owing to an over-dose of X-linked genes in extra-embryonic tissues [2,3,10]. Notably, at late preimplantation phases, the imprinted Xm-*Xist* silencing (Xm-*Xist* imprinting) is partially disrupted in parthenogenetic embryos, which have two maternal X-chromosomes (XmXm) [4,11]. A previous study showed that histone 3 lysine 9 trimethylation (H3K9me3) and/or histone acetylation was involved in Xm-*Xist* derepression from early preimplantation phases [4]. However, little is known about the molecular mechanism underlying Xm-*Xist* imprint maintenance or loss in fertilized (male: XmY or female: XmXp) or XmXm embryos (parthenogenotes), respectively. Furthermore, the question of whether transient alteration of histone modifications from early preimplantation phases could lead to stable Xm-*Xist* derepression in postimplantation stages has not previously been addressed.

In this study, we demonstrated that chromatin condensation at Xm-*Xist/Tsix* genomic loci was essential for Xm-*Xist* silencing in early preimplantation phases. This condensation was involved in H3K9me3 and histone acetylation. Once the chromatin was decondensed in early preimplantation phase, Xm-*Xist* was stably derepressed and resulted in the rescue of female lethality by *Xist* paternal deletion (XmXp^Δ^), indicating that loss of Xm-*Xist* imprinting was irreversible and genetic lethality could be overcome without direct gene manipulation. At late preimplantation phases, Rnf12 dosage played an important role in Xm-*Xist* silencing in fertilized and parthenogenetic embryos. Furthermore, we found Oct4 served as a chromatin opener to create transcriptional permissive states at Xm-*Xist*/*Tsix* genomic loci in fertilized and parthenogenetic embryos. Together, we propose that *Xist* imprinting maintenance depends on chromatin condensation states and the regulators differ at developmental stage.

### Chromatin decondensation at Xm-*Xist*/*Tsix* loci through loss of H3K9me3 and gain of histone acetylation in early preimplantation phases

**Fig 1:**
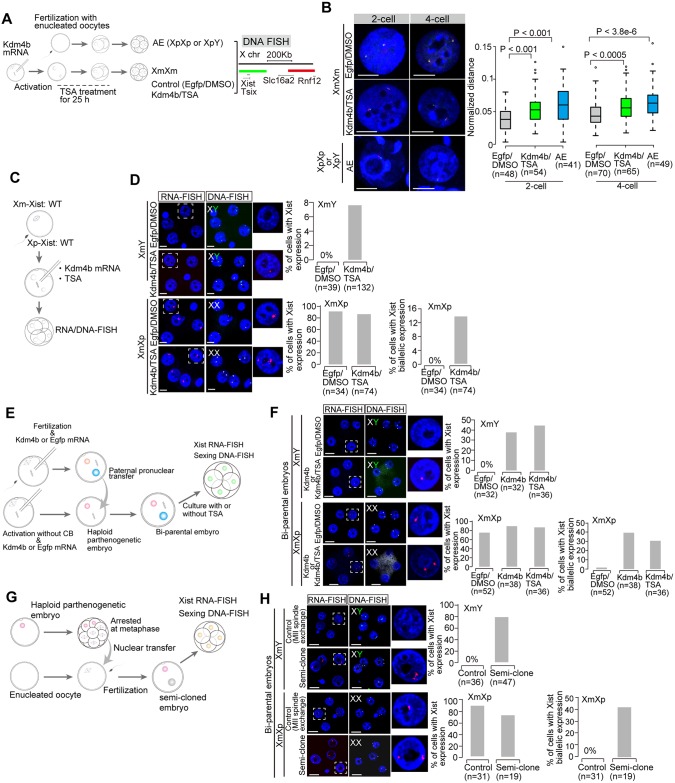
Chromatin decondensation induced by loss of H3K9me3 and gain of histone acetylation. (a) Experimental scheme for chromatin condensation assay using DNA-FISH. Each colour corresponds to the FISH image. Androgenetic embryos (XpXp or XpY) were produced by *in vitro* fertilization with enucleated oocytes. For the production of Kdm4b/TSA- or Egfp/DMSO-XmXm embryos, mRNA was injected into MII oocytes and then the oocytes were activated by SrCl_2_. (b) DNA analysis in control (*Egfp* mRNA with DMSO treatment: Egfp/DMSO)-XmXm, Kdm4b/TSA-XmXm, and androgenetic embryos at 2-cell and 4-cell stages. Representative images and the values of the normalized distance between two signals are shown as pictures and graphs, respectively. The P-values were calculated by the Mann-Whitney U test. (c) Experimental scheme of bi-parental embryos production with *Kdm4b* and TSA treatment. Oocytes were subjected to *in vitro* fertilization and *Kdm4b* mRNA was injected at 1.5 hours after insemination. The *Kdm4b*/TSA-treated fertilized embryos were cultured and analyzed at 4-cell stage by RNA/DNA-FISH. The sexing probe for X chromosome detection targets the XqF4 regions. (d) RNA/DNA-FISH analysis in Egfp/DMSO- or Kdm4b/TSA-XmY and -XmXp embryos at the 4-cell stage. The graph shows quantification of RNA-FISH signals. n, the number of cells analysed. Scale bars show 20 μm. (e) Experimental scheme of bi-parental embryo production. Oocytes were subjected to *in vitro* fertilization and *Kdm4b* mRNA was injected at 1.5 hours after insemination. Haploid parthenogenetic embryos (hPE) injected with *Kdm4b* mRNA were produced by SrCl_2_-mediated activation without cytochalasin B (CB). *Egfp* mRNA was used as an injection control. At 6 hours after insemination or activation, a paternal pronucleus that was larger than the maternal pronucleus in the fertilized embryo was transferred into hPE to produce a bi-parental embryo. The bi-parental embryos were subjected to *Xist* RNA-FISH and sexing by DNA-FISH at the 4-cell stage. (f) RNA/DNA-FISH analysis of bi-parental embryos. Representative images of bi-parental embryos with Kdm4b/TSA or Kdm4b. The graph shows quantification of RNA-FISH signals. n, the number of cells analysed. Scale bars show 20 μm. (g) Experimental scheme of semi-cloned embryo production. hPE were cultured at the morula stage and the nuclei were arrested by nocodazol treatment. The metaphase-arrested nuclei were transferred into enucleated oocytes and the constructed oocytes were subjected to fertilization, resulting in semi-cloned embryos. For control embryos, spindles were exchanged between oocytes. (h) RNA/DNA-FISH analysis of semi-cloned embryos. Representative images of semi-cloned and control embryos. The graph shows quantification of RNA-FISH signals. n, the number of cells analysed. Scale bars show 20 μm.

A previous study using XmXm embryos (parthenogenotes) showed that loss of H3K9me3 via Kdm4b, which is a H3K9me3 demethylase [12], or gain of histone acetylation by trichostatin A (TSA) treatment, induced Xm-*Xist* derepression [4] (S1 Fig). More recently, chromatin decondensation was shown to be associated with *Xist* expression [13]. Thus, we first investigated whether the chromatin condensation states of Xm-*Xist*/*Tsix* regions at the 2- and 4-cell stages could be altered by Kdm4b overexpression and TSA treatment using XmXm embryos (Kdm4b/TSA-XmXm) (Fig 1a). We also examined the chromatin states of androgenetic embryos with *Xist* RNA positive alleles [14]. DNA fluorescence *in situ* hybridization (DNA-FISH) analysis around *Xist*/*Tsix* genomic regions revealed that Kdm4b/TSA-XmXm embryos showed significantly relaxed chromatin states in both stages compared with Egfp/DMSO (control)-XmXm embryos, although the chromatin was the most relaxed in Xp at both stages (Fig 1b).

We next examined whether Kdm4b/TSA treatment could induce Xm-*Xist* derepression in XmXp and XmY embryos (fertilized embryos), respectively, at the 4-cell stage (Fig 1c). RNA combined with DNA-FISH (RNA/DNA-FISH) analysis showed that Xm-*Xist* derepression was observed in 7.6% of Kdm4b/TSA-XmY cells and that 13.5% of Kdm4b/TSA-XmXp cells showed biallelic expression (Fig 1d). Thus, these results indicated that the loss of H3K9me3 and gain of histone acetylation induced chromatin decondensation at Xm-*Xist*/*Tsix* genomic regions, resulting in Xm-*Xist* derepression.

Although these results indicated that the chromatin alterations induced Xm-*Xist* derepression in XmY and XmXp embryos, the induction efficiency was low compared with XmXm embryos (Fig 1d and S1b Fig). In comparison, a previous study showed that the sole induction of *Kdm4b* mRNA sufficiently induced Xm-*Xist* derepression in XmXm 4-cell embryos [4]. Notably, it has been shown that the transcriptional capacity of maternal pronuclei in haploid parthenogenetic embryos (hPE) was higher than that of paternal and maternal pronuclei in zygotes [15]. Furthermore, although histone H4 acetylation was predominantly imposed on the paternal pronuclei in zygotes, maternal pronuclei in parthenogenetic embryos exhibited an H4 acetylated state [16]. These results suggested that the absence of the paternal genome during pronuclear formation might provide a transcriptionally permissive state within the maternal genome. Consistent with this notion, very few XmXm embryos showed Xm-*Xist* derepression at the 4-cell stage, although Xm-*Xist* was never expressed in XmY and XmXp embryos [4] (S1b Fig).

In light of these findings, we speculated that the presence of paternal genome during pronuclear formation would impede Xm-*Xist* derepression. In order to inspect the possibility, we constructed Kdm4b overexpressing bi-parental embryos wherein the parental pronuclei were of different derivation: the maternal pronucleus was formed by SrCl_2_ activation whereas the paternal pronucleus was formed by *in vitro* fertilization. Then, to produce bi-parental embryos, paternal pronuclei were transferred into haploid maternal embryos derived from SrCl_2_ activation (Fig 1e). At the 4-cell stage, *Xist* RNA-FISH analysis revealed that the constructed bi-parental embryos with Kdm4b overexpression showed marked increase of the cells with Xm-*Xist* derepression in XmY embryos (Fig 1f: 37.5%; 4.9 fold compared with Kdm4b/TSA-XmY in Fig 1d) and with bialleleic *Xist* expression in XmXp embryos (Fig 1f: 39.5%; 2.9 fold compared with Kdm4b/TSA-XmXp in Fig 1d), whereas control embryos showed no Xm-*Xist* derepression in XmY embryos and only a cell was biallelic expression in XmXp embryos (2%) (Fig 1f). The combination of TSA with *Kdm4b* mRNA injection was also able to induce Xm-*Xist* derepression (Fig 1f). Thus, these results indicated that the presence of the paternal genome during pronuclear formation impeded Xm-*Xist* derepression by chromatin alterations.

However, the question remained whether Xm-*Xist* could be derepressed irrespective of the presence of the paternal genome during pronuclear formation when the maternal chromatin was sufficiently decondensed before fertilization. To test this, we constructed oocytes with decondensed maternal chromatin derived from hPE at the morula stage (Fig 1g), because a previous study had indicated that Xm-*Xist*/*Tsix* genomic regions in XmXm embryos became gradually relaxed during preimplantation phases [13]. The constructed oocytes were subjected to fertilization and resulted in semi-cloned embryos (Fig 1g). At the 4-cell stage, *Xist* RNA-FISH analysis revealed that 78.7% of the cells in XmY semi-cloned embryos exhibited Xm-*Xist* derepression (Fig 1h: 10.3-fold increase compared with Kdm4b/TSA-XmY in Fig 1c) and 36.8% of cells in XmXp semi-cloned embryos showed biallelic expression (Fig 1h: 2.7-fold increae compared with Kdm4b/TSA-XmXp in Fig 1c). In contrast, in control embryos (spindle-exchanged oocytes), we did not observed Xm-*Xist* derepression in XmY embryos or biallelic expression in XmXp embryos (Fig 1h).

Taken together, these results demonstrated that Xm-*Xist* could be derepressed if the chromatin was decondensed even when the paternal genome was present during pronuclear formation, indicating that chromatin condensation of the maternal genome represents the primary factor for imprinting maintenance to silence Xm-*Xist*. The condensation could be relaxed by loss of H3K9me3 and gain of histone acetylation.

### Xm-*Xist* derepression causes global silencing of X-linked genes in XmXp^Δ^ embryos

**Fig 2:**
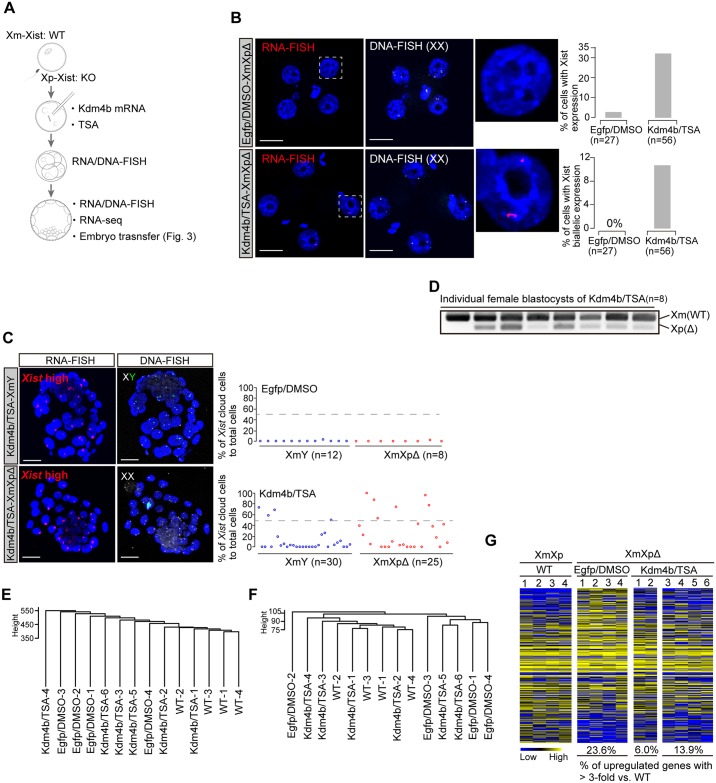
Xm-*Xist* induces global X-linked gene silencing in XmXp^Δ^ embryos. (a) Experimental scheme to construct Kdm4b/TSA-XmXp^Δ^ embryos. (b) RNA/DNA-FISH analysis in Kdm4b/TSA- or Egfp/DMSO-XmXp^Δ^ embryos at the 4-cell stage. The graph shows quantification of RNA-FISH signals. The upper and lower graphs show the percentages of *Xist*-expressing and biallelic cells, respectively. n, the number of cells analysed. Scale bars show 20 μm. (c) RNA/DNA-FISH analysis in Kdm4b/TSA or control blastocysts. Each circle shows individual embryos. n, the number of embryos analysed. Scale bars show 20 μm. (d) Strand-specific RT-PCR analysis for *Xist* detection. Individual Kdm4b/TSA-XmXp^Δ^ blastocysts were used for the assay. (e and f) Hierarchical clustering analysis by RNA-Seq from individual blastocysts of WT-XmXp, Egfp/DMSO-XmXp^Δ^, and Kdm4b/TSA-XmXp^Δ^. The genes expressed in at least one sample (TMM > 10) were used for analysis. All genes expressed (e) and X-linked genes (f). (g) Heat map showing X-linked genes expression. Colours show expression levels; blue: low, black: middle, and yellow: high. The percentages are the genes with > 3-fold upregulated compared with WT.

Next, we asked whether the derepression of Xm-*Xist* during the early preimplantation stages could be stably maintained. To facilitate the analysis of Xm-*Xist* derepression state in female embryos, we used female embryos devoid of Xp-Xist expression because of a paternal deletion in the repeat-A region [10] (Fig 2a). We first checked *Xist* expression states by RNA/DNA-FISH analysis at the 4-cell stage, demonstrating that 32.1% of Kdm4b/TSA-XmXp^Δ^ cells but only one Egfp/DMSO-XmXp^Δ^ cell (3.7%) exhibited an *Xist* signal (Xist+) (Fig 2b). Furthermore, 10.7% of Kdm4b/TSA-XmXp^Δ^ cells showed biallelic expression (Fig 2b). Given that Egfp/DMSO-XmXp^Δ^ embryos showed no *Xist* cloud and biallelic expression, the results indicated that histone modification alteration induced *Xist* expression on not only Xm but also Xp^Δ^ alleles.

We further examined whether Xm-*Xist* derepression could be stably maintained through late preimplantation stages. In blastocysts, RNA/DNA-FISH showed that 13.3% of Kdm4b/TSA-XmY and 24% of Kdm4b+TSA-XmXp^Δ^ embryos exhibited robust *Xist* expression states (>50% of cells), whereas no *Xist* expression was found in *Egfp*/DMSO treated embryos (Fig 2c). We also examined the effect of Kdm4b induction alone on *Xist* expression. Although *Xist* expression was induced in XmXp^Δ^ embryos, no embryos of either gender exhibited >50% of *Xist* positive cells (S2 Fig), indicating that both H3K9me3 loss and the absence of histone deacetylases were required for strong *Xist* induction.

The antisense RNA for *Xist*, commences around the blastocysts stage [17,18]. Therefore, to investigate allele-specific *Xist* expression, we performed strand-specific reverse transcription polymerase chain reaction (RT-PCR) analysis. This demonstrated that Xm-*Xist* expression was clearly induced in Kdm4b/TSA-XmXp^Δ^ embryos (Fig 2d). Thus, Xm-*Xist* derepression at early preimplantation phases could last until the late phases of preimplantation.

To further examine *Tsix* expression states, we performed RNA/DNA-FISH analysis using *Tsix*-specific detection probes (S3a Fig). Kdm4b/TSA treatment resulted in an increase of cells with *Xist* but not *Tsix* in XmY and XmXp embryos (S3b and S3c Fig). Quantitative PCR (qPCR) analysis also confirmed the lack of *Tsix* upregulation although some X-linked genes, *i*.*e*., *Pgk1* and *Plac1*, were downregulated in Kdm4b/TSA-XmY and -XmXp^Δ^ embryos compared with Egfp/DMSO treated embryos (S3d and S3e Fig). We also confirmed H3K27me3 enrichment in some cells in Kdm4b/TSA-XmY or -XmXp^Δ^ blastocysts [19], indicating that the normal XCI process occur in Kdm4b/TSA treated embryos (S3f Fig).

To gain further insights into transcriptome states, we performed RNA deep sequencing (RNA-Seq) analysis using an individual XmXp^Δ^ embryo with Kdm4b/TSA, Egfp/DMSO, and wild-type (WT). Notably, hierarchical clustering analysis indicated that the transcriptome states of two Kdm4b/TSA-XmXp^Δ^ (Kdm4b/TSA-1/2) embryos resembled those of WT (Fig 2e), indicative of normal X-linked genes expression in the Kdm4b/TSA-1/2 embryo. Consistent with this, hierarchical clustering based on X-linked genes showed that Kdm4b/TSA-1/2 clustered with WT embryos (Fig 2f). Out of 331 X-linked genes expressed, 23.6% and 13.9% were upregulated in Egfp/DMSO and Kdm4b/TSA-3/4/5/6 embryos, respectively (Fig 2g and S1 Table). However, only, 6.0% were upregulated in Kdm4b/TSA-1/2 embryos (Fig 2g and S1 Table). Although group specific differentially-expressed genes were also identified (S4 Fig), these results indicated that Kdm4b/TSA treatment induced Xm-*Xist* derepression and reduced the number of upregulated X-linked genes in XmXp^Δ^ embryos.

### Xm-*Xist* compensates for imprinted XCI

**Fig 3:**
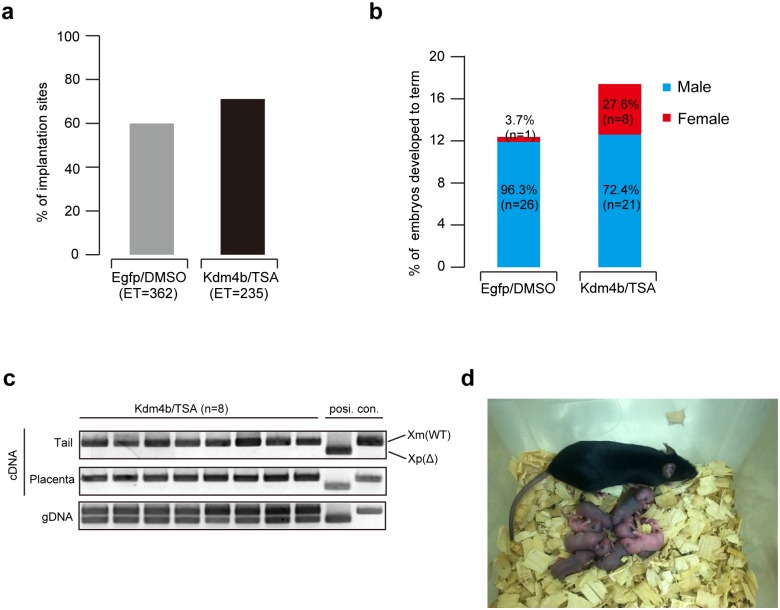
Developmental competency of Kdm4b/TSA-XmXp^Δ^ embryos. (a) % of implantation sites at E19.5. n, the number of transferred embryos. (b) % of embryos developed to term at E19.5. n, the number of recovered embryos. (c) PCR analysis using cDNA and genomic DNA (gDNA) in rescued XmXp^Δ^ females. cDNA from the tails and placentas and gDNA from placentas were used for the assay, respectively. (d) The rescued females developed to adults with normal reproductive ability.

To test whether transient histone alterations in preimplantation phases could lead to stable Xm-*Xist* derepression in embryonic- and extraembryonic tissues and rescue the lethal phenotype of XmXp^Δ^ embryos without gene manipulation, we conducted embryo transfer experiments and assessed the developmental ability of XmXp^Δ^ embryos. We transferred 362 Egfp/DMSO- and 235 Kdm4b/TSA-blastocysts that were recovered at embryonic day 19.5, identifying 60% and 71% of implantation sites in Egfp/DMSO- and Kdm4b/TSA-groups, respectively (Fig 3a). We obtained 26 XmY pups (96.3% of total pups) from the Egfp/DMSO treatment. Unexpectedly, one XmXp^Δ^ pup was also born in the group (Fig 3b). However, we have not yet replicated this result. In contrast, 8 XmXp^Δ^ (27.6%) and 21 XmY (72.4%) pups were born in the Kdm4b/TSA group (Fig 3b).

RT-PCR analysis of embryonic- and extra-embryonic tissue from rescued XmXp^Δ^ embryos exhibited predominant Xm-*Xist* expression (Fig 3c). The rescued females displayed normal reproduction and gave birth to viable offspring (Fig 3d). Taken together, these results clearly demonstrated that the Xm-*Xist* compensated for imprinted XCI and exhibited the functional equivalency of Xm-*Xist* to Xp-*Xist* in both embryonic- and extraembryonic tissues.

### Decrease of Xm-*Tsix* expression in XmXm embryos compared with that in XmXp and XmY embryos

One of the remaining questions is the molecular mechanisms involved in loss of the Xm-*Xist* imprint (Xm-*Xist* silencing) in XmXm morula embryos despite the imprint maintenance in XmY and XmXp embryos [4,11]. As the chromatin at *Xist/Tsix* genomic loci was gradually relaxed during preimplantation development [13] and Xm-*Tsix* began to be expressed around the morula stage [17], we investigated the *Tsix* expression state. RNA-FISH analysis for *Xist* and *Tsix* revealed that *Tsix* was also not detected until the 16-cell stage in XmXp, XmY, and XmXm embryos. In XmXp embryos, the cells showing a *Tsix* signal or an *Xist* cloud averaged 23% and 94%, respectively (S5 Fig). XmY embryos contained 34% of cells with *Tsix* expression and no *Xist* expression (S5 Fig). In XmXm embryos at the 16-cell stage, an *Xist* cloud was observed in 34% of nuclei. Notably, the ratio of *Tsix* expressing cells in XmXm embryos was less than that in XmXp and XmY embryos (S5 Fig, XmXm: 18%). Considering that Xm-*Tsix* was present in two copies in XmXm embryos, these results implied that Xm-*Xist* derepression in XmXm embryos might be associated with the repression of *Tsix*.

### *Rnf12* is overexpressed and induces Xm-*Xist* activation via *Tsix* repression in XmXm embryos

**Fig 4:**
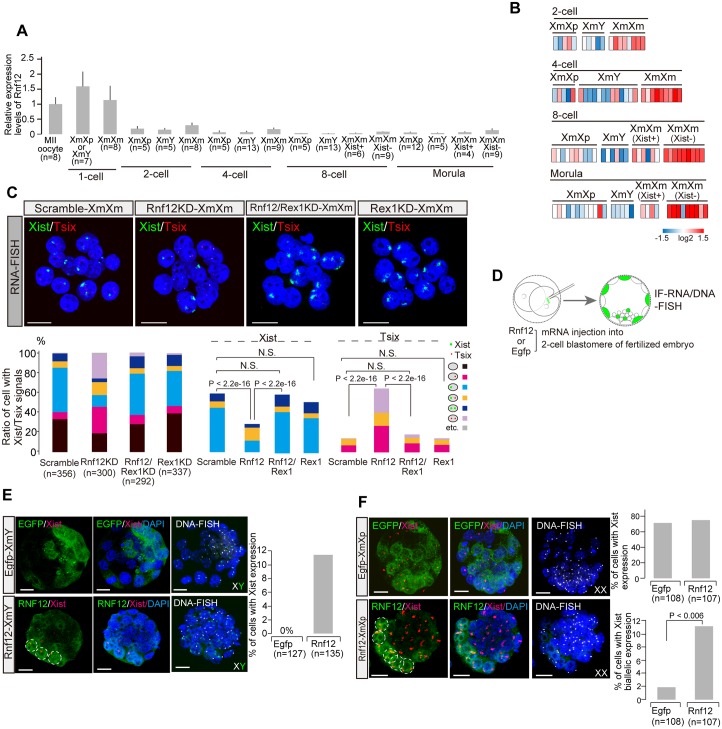
*Rnf12* over-dosage induces Xm-*Xist* derepression in XmXm, XmY and XmXp embryos. (a) Average *Rnf12* expression levels in single XmY, XmXp, and XmXm cells from oocytes to morula embryos. n, the number of cells analysed. The average expression levels of MII oocytes were set as one. (b) *Rnf12* expression profiles in individual cells. The average expression levels of XmXp embryos were set as one in each stage. (c) *Xist*/*Tsix* RNA-FISH analysis in Rnf12KD-XmXm and Rex1/Rnf12 double KD XmXm morulae. Representative images from scramble (siRNA control), Rnf12KD, and Rex1/Rnf12 double KD embryos. The graphs show quantification of FISH signal patterns. The P-values were calculated by the Fisher’s exact test. n, the number of cells analysed. Scale bars show 20 μm. (d) Experimental scheme of construction of RNF12 overexpressing fertilized embryos by *Rnf12* mRNA injection. *Egfp* mRNA was used for the control. (e and f) Immunofluorescence combined with RNA/DNA-FISH analysis of Rnf12 overexpressing XmY (Rnf12-XmY) and control XmY (Egfp-XmY) (e), Rnf12-XmXp, and Egfp-XmXp (f). Representative images from *Rnf12* or *Egfp* overexpressing embryos. The graph shows % of *Xist* cloud cells in XmY and XmXp embryos or of *Xist* biallelic cells. n, the number of cells expressing RNF12 or EGFP. Scale bars show 20 μm.

Previous studies indicated that the dose-dependent *Xist* activator, RNF12 [6,7], was highly expressed in early preimplantation phases [4]. RNF12 activates *Xist* via REX1 protein degradation and REX1 plays a role in *Tsix* elongation [20,21]. Thus, we performed single cell qPCR assays against *Rnf12* mRNA using XmY, XmXp, and XmXm preimplantation embryos. To identify the sex of the cells in fertilized embryos at the 2-cell stage, DNA-FISH analysis was conducted in the remaining cell in each embryo not used for qPCR analysis. From the 4-cell stage onward, *Xist* expressing cells were defined as female. The analysis exhibited that *Rnf12* expression levels were markedly higher in oocytes and at the 1-cell stage (Fig 4a). Although *Rnf12* expression levels gradually decreased in most of the embryos as the embryos developed, the levels of XmXm (*Xist*−) cells tended to be high compared with those of XmXp from the 2-cell stage onward (Fig 4b). At 8-cell and morula stages, the *Rnf12* levels of XmXm (*Xist*+) cells were downregulated compared with XmXm (*Xist*−) and XmXp (≥ 2 fold on average) (Fig 4b). These results suggested that excess *Rnf12* might facilitate Xm-*Xist* derepression via Xm-*Tsix* repression in XmXm embryos.

To test this possibility, we constructed Rnf12-depleted XmXm embryos by siRNA injection (Rnf12KD-XmXm) (S6a and S6b Fig). The RNA-FISH analysis for Xm-*Xist*/*Tsix* revealed that *Rnf12* repression caused a remarkable increase of *Tsix*+ (scramble-XmXm: 14% vs. Rnf12KD-XmXm: 64%, Fig 4c), and the proportion of *Xist*+ cells significantly declined (scramble-XmXm: 59% vs. Rnf12KD-XmXm: 28%, Fig 4c). Next, we tested the previous notion in differentiating ES cells, which indicated that RNF12-mediated *Xist* upregulation was involved in the *Rex1* expression state [20], as determined by *Rnf12*/*Rex1* double knockdown (KD) experiments in XmXm embryos (S6c Fig). As expected, the Xm-*Xist* repression with *Tsix* upregulation seen in Rnf12KD-XmXm embryos was rescued in XmXm embryos with *Rnf12*/*Rex1* double depletion, although there was no marked effect on Xm-*Xist/Tsix* expression in *Rex1* single KD embryos (Fig 4d). Thus, we concluded that the *Rnf12* overdose in XmXm embryos caused Xm-*Tsix* repression and resulted in Xm-*Xist* activation.

### Additional RNF12 expression induces Xm-*Xist* activation in XmY and XmXp embryos

Given the above results from XmXm embryos, we inferred that the decrease of *Rnf12* in XmY and XmXp embryos around morula stages might be essential for Xm-*Tsix* activation to repress Xm-*Xist* since the chromatin was decondensed. To test this possibility, we constructed Rnf12-overexpressing fertilized embryos. As RNF12 turnover was implied to be quick [4], we injected *Rnf12* mRNA into 2-cell blastomeres (Fig 4d). At the blastocyst stage, we carried out immunofluorescence against RNF12 combined with RNA/DNA-FISH (IF-RNA/DNA-FISH). Of the RNF12 overexpressing cells in XmY, 11.1% exhibited *Xist* cloud states (Fig 4e), whereas embryos with *Egfp* mRNA never showed Xm-*Xist* derepression (Fig 4e). In XmXp embryos, biallelic expression was significantly induced in *Rnf12* overexpressing cells (*Egfp*: 1.9% vs. *Rnf12* overexpression: 11.2%, Fig 4f). These results indicated that proper *Rnf12* expression levels were required for Xm-*Xist* silencing in fertilized embryos.

### Oct4 positively controls Xm-*Xist*/*Tsix* in XmXm embryos at late preimplantation phases

Around the morula stage, many pluripotency-factors that were shown to regulate *Xist* begin to be expressed [21–23]. Therefore, using XmXm morulae, we investigated the involvement of pluripotency-factors in Xm-*Xist*/*Tsix* regulation. YY1 and Oct4 were selected for candidate pluripotency-factors based on previous reports [23–25] and we conducted siRNA-mediated KD experiments. qPCR and *Xist* RNA-FISH analysis revealed that *Oct4* depletion induced significant reduction of Xm-*Xist* (S7 Fig), implying that Oct4 is an important factor for regulating Xm-*Xist* imprint erasure.

**Fig 5:**
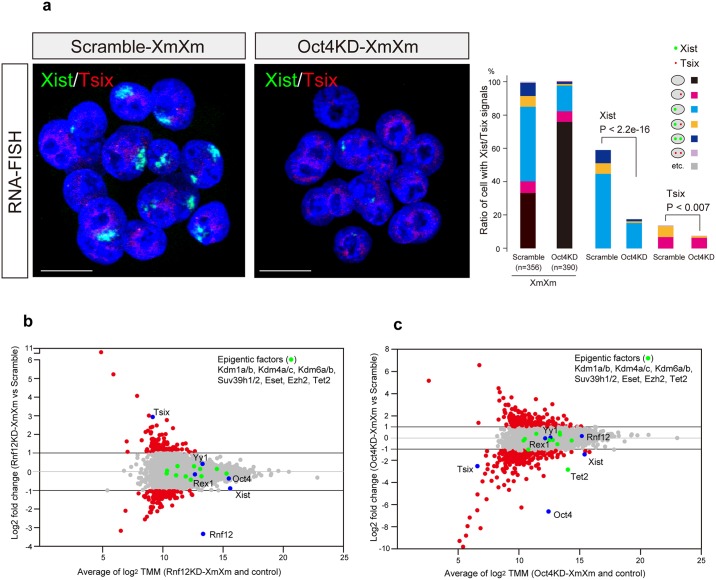
*Oct4* depletion disrupts Xm-*Xist*/*Tsix* expression in XmXm embryos. (a) *Xist*/*Tsix* RNA-FISH analysis in Oct4KD-XmXm and scramble-XmXm morulae. Scramble data is the same as in Fig 4c. The graphs show quantification of FISH signal patterns. The P-values were calculated by the Fisher’s exact test. n, the number of cells analysed. Scale bars show 20 μm. (b and c) MA plot of RNA-Seq analysis in Rnf12KD-XmXm embryos (b) and Oct4KD-XmXm embryos (c). Red circles show fold change over 2-fold. Representative epigenetic markers and known *Xist/Tsix* regulators are depicted as green and blue, respectively.

Further, *Xist*/*Tsix* RNA-FISH analysis in Oct4KD-XmXm embryos revealed that the proportions of cells with *Xist* and *Tsix* signals were extremely reduced (Oct4KD-XmXm: *Xist* 18% and *Tsix* 8%, Fig 5a and scramble: *Xist* 59% and *Tsix* 14%, shown in Fig 4c), indicating that opposed to RNF12, Oct4 controls not only Xm-*Xist* derepression but also Xm-*Tsix* activation.

To gain further insights into the effect of *Oct4* and *Rnf12* depletion in XmXm morulae, we performed RNA-Seq analysis. Out of 7898 genes with > 10 trimmed mean of M values (TMM) [26] in at least one group, 280 and 613 genes were differentially expressed (more than 2-fold) in Rnf12KD-XmXm and Oct4KD-XmXm embryos, respectively (Fig 5b and 5c and S2 Table), indicating the high impact of *Oct4* depletion on the transcriptome. Notably, the differentially expressed genes following *Oct4* or *Rnf12* depletion were randomly distributed across the chromosomes (S8 Fig).

Consistent with the FISH results, *Xist* and *Tsix* were down- and upregulated in Rnf12KD-XmXm embryos, respectively (Fig 5b and S2 Table), whereas both were repressed in Oct4KD-XmXm embryos (Fig 5c and S2 Table). *Oct4* and *Rnf12* expression levels were comparable to those of scramble-XmXm embryos in Rnf12KD-XmXm and Oct4KD-XmXm embryos, respectively (Fig 5b and 5c and S2 Table). The expression states of major histone modifiers including members of the H3K9me3 demethylase *Kdm4*-family were not dramatically affected (Fig 5b and 5c and S2 Table). However, we found that *Tet2*, which is associated with DNA methylcytosine dioxygenase [27], was markedly downregulated (14% of scramble) only in Oct4KD-XmXm morulae (Fig 5c and S2 Table). To examine the impact of *Tet2* depletion on Xm-*Xist* derepression, we constructed Tet2KD-XmXm embryos and evaluated their Xm-*Xist* expression states. RNA-FISH analysis indicated that the extent to which *Tet2* mediated Xm-*Xist* repression was modest compared with *Oct4* depletion (S9 and S7c Figs). However, as we could not exclude the possibility that the *Tet2* KD efficiency might be insufficient, given that DNA methylation was not responsible for Xm-*Xist* expression [28], these results suggested that dysregulation of epigenomic factors were not likely to be the primary cause for Xm-*Xist* repression following *Oct4* depletion.

The known *Xist* activators on the X chromosome (*Jpx* and *Ftx*) [29,30] were not detectable in either group by qPCR analysis. Taken together, these results indicated that the mechanism by which Oct4 mediated *Xist* regulation was different from that underlying Rnf12-mediated regulation.

### *Oct4* directs chromatin decondensation at Xm-*Xist*/*Tsix* loci in XmY, XmXp, and XmXm embryos

**Fig 6:**
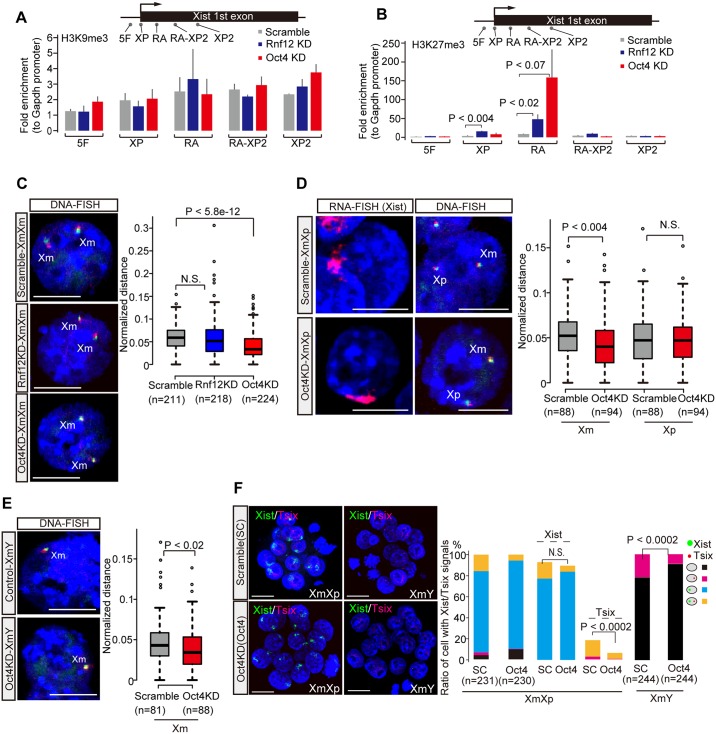
Oct4 directs chromatin decondensation at Xm-*Xist*/*Tsix* regions in XmY, XmXp, and XmXm embryos. (a) H3K9me3 and H3K27me3 states around *Xist* regulatory regions in XmXm morulae. Examined regions for eChIP-qPCR in XmXm morula embryos were shown above the graph. The XP region is major promoter. H3K9me3 (a) and H3K27me3 (b) states. In all cases, more than three biological replicates were tested. The error bars show standard error. The P-values were calculated using the Student’s t-test. (c) DNA-FISH analysis in Oct4KD- and Rnf12KD-XmXm morulae. (d and e) RNA/DNA-FISH analysis in Oct4KD-XmY and -XmXp morulae. *Xist* cloud cells by RNA-FISH were identified as Xp alleles. BAC DNA probes shown in Fig 1a were used for the DNA-FISH assay (c-e). Representative images and the values of normalized distance between two signals are shown as pictures and graphs, respectively. The P-values were calculated by the Mann-Whitney U test. Scale bars show 10 μm. (f) *Xist*/*Tsix* RNA-FISH analysis in Oct4KD-XmY and -XmXp morulae. The graphs show quantification of FISH signal patterns. The P-values were calculated by the Fisher’s exact test. Scale bars show 20 μm. n, the number of cells analysed.

Previous studies demonstrated that H3K9me3 was involved in Xm-*Xist* silencing and that *Tsix* transcripts altered H3K27me3 states at *Xist* promoter regions [31,32]. As such, we investigated the two histone modifications at Xm-*Xist* promoter regions in Oct4KD-XmXm and Rnf12KD-XmXm embryos using embryo-chromatin immunoprecipitation combined qPCR [4,13]. Notably, we found nosignificant changes of H3K9me3 modifications in Oct4KD- and Rnf12KD-XmXm embryos compared with scramble-XmXm embryos in the regions examined (Fig 6a) whereas, as expected, in Rnf12KD-XmXm embryos, significant hypermethylation of H3K27me3 at the promoter regions compared with scramble-XmXm embryos was observed (4.7-fold increase, Fig 6b). Oct4KD-XmXm embryos also showed this effect, albeit more modest, (2.4-fold increase compared to scramble-XmXm, Fig 6b). The repeat-A regions were also markedly hypermethylated in *Rnf12* or *Oct4* depleted XmXm embryos (Rnf12KD: 5.5 fold and Oct4: 18.4 fold increase compared to scramble-XmXm, respectively, Fig 6b). Thus, these results indicate that *Oct4* and *Rnf12* were involved in the alteration of histone modifications leading to transcriptional active states around *Xist* regulatory regions.

Since *Oct4* repression caused not only Xm-*Xist* but also Xm-*Tsix* silencing in XmXm embryos, we inferred that Oct4 might also regulate chromatin condensation states at Xm-*Xist*/*Tsix* genomic regions. To test this possibility, we conducted DNA-FISH analysis in XmXm morulae. Chromatin condensation at the loci was significantly induced by *Oct4* depletion but was not observed upon *Rnf12* repression (Fig 6c). These results indicated that Oct4 mediated chromatin relaxation facilitated transcription around *Xist*/*Tsix* regions and resulted in Xm-Xist/*Tsix* activation in XmXm embryos.

Next, we investigated whether Oct4 served as chromatin opener in XmXp and XmY embryos. To distinguish Xp and Xm alleles, we conducted RNA/DNA-FISH at the morula stage. Notably, *Oct4* depletion significantly induced chromatin contraction in both XmY and XmXp embryos at Xm-*Xist*/*Tsix* (Fig 6d and 6e). Given this, we sought to investigate whether Oct4 might affect Xm-*Tsix* expression states, by *Xist*/*Tsix* RNA-FISH analysis in Oct4KD-XmY and -XmXp embryos. Notably, *Xist* expression states in XmXp embryos were comparable between scramble and Oct4KD embryos (Fig 6f), indicating that Oct4 did not affect Xp-*Xist* expression. However, as expected, Oct4KD embryos exhibited a significant reduction of cells with *Tsix*+ in both XmY (9.0%) and XmXp (6.1%) compared to scramble-XmY (21.7%) and -XmXp (18.2%) cells counterparts (Fig 6f). Taken together, these results revealed the novel role of Oct4 as a chromatin opener to induce the activation of Xm-*Xist*/*Tsix* in XmXm and of Xm-*Tsix* in XmY and XmXp embryos.

## Discussion

**Fig 7:**
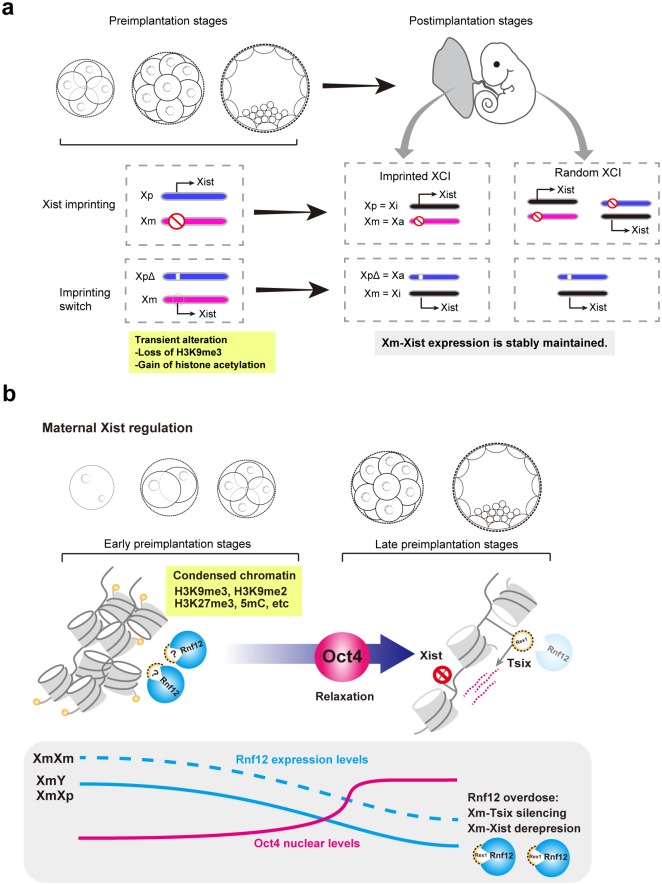
Proposed model. (a) Irreversible Xm-*Xist* imprinting. The transient alteration of histone modifications through loss of H3K9me3 and gain of histone acetylation induces stable Xm-*Xist* derepression and results in the rescue of lethality in XmXp^Δ^ without gene manipulation. The imprinting switch in XCI does not affect cellular integrity. (b) Maintenance and erasure of the Xm-*Xist* imprint. In early preimplantation phases, chromatin at Xm-*Xist*/*Tsix* regions is condensed by various epigenetic modifications. At late stage, Oct4 localizes to nucleus (see discussion) and serves as a chromatin opener at Xm-*Xist*/*Tsix* regions, creating transcriptional permissive states around *Xist*/*Tsix* regions. In XmXm embryos, *Rnf12* expression levels represent a double dose compared with those of XmY or XmXp embryos, leading to Xm-*Xist* activation by *Tsix* silencing, which depends on REX1 state. In XmY and XmXp embryos, on the other hand, physiological expression levels of *Rnf12* are essential for Xm-*Xist* silencing by Xm-*Tsix* activation.

The establishment of XCI is crucial for faithful development [2,3]. The present study addressed two unresolved questions about imprinted XCI in mice, the first being the irreversibility of Xm-*Xist* imprinting. Once Xm-*Xist* was derepressed at early preimplantation phases by transient alteration of histone modifications, it could be stably maintained and genetic lethality of XmXp^Δ^ embryos could be rescued without gene manipulation (Fig 7a). The other is the molecular mechanism of imprinted XCI maintenance and erasure. The maintenance mechanism of Xm-*Xist* imprinting differed by developmental phase: in early preimplantation phases, chromatin condensation states determined the Xm-*Xist* silencing, whereas in the late preimplantation phase, as chromatin was relaxed by Oct4, the occurrence of maintenance or erasure of the *Xist* imprinting depended on *Rnf12* dosage state (Fig 7b).

### Chromatin condensation and imprinted XCI

Species-specific imprinted XCI has been observed and one study indicated that human embryos showed no imprinted XCI [33]. Previously, Sado and Sakaguchi proposed that chromatin condensation states in parental genomes differed in each specie and that this might define imprinted XCI [34]. In the current study, we showed that the asymmetric chromatin condensation states of parental *Xist*/*Tsix* genomic regions are crucial for the initiation of *Xist* expression in mice (Fig 7b). In mice, the Xm-*Xist* imprint is established during oogenesis [13,35]. During the phases, a maternal genome state is imposed on many transcriptionally repressive marks such as 5mC of DNA, H3K27me3, and H3K9me2/3 [4,36–38]. Furthermore, HDAC2, which mediates induced histone deacetylation, is not highly expressed until the full grown oocyte stage [39]. Thus, maternal chromatin becomes condensed during oocyte growth [13]. In mice, the maternal pronucleus is smaller than its paternal counterpart after fertilization, reflective of the maternal genome condensation. As a reflection of the chromatin condensation states in the maternal genome immediately after fertilization, the maternal pronuclear size is smaller than paternal size in mice [4], whereas in humans, the parental pronuclei size was equal and non-imprinted *XIST* expression was observed [40].

### Oct4-mediated chromatin decondensation

Our findings disclosed a novel role of Oct4 in *Xist* regulation *in vivo*. Maternal *Oct4* has been shown to be dispensable for embryonic development [41]. Recently, we found that the Oct4 protein was not localized to the nucleus until the 8–16-cell stage in mice [42]. Moreover, Oct4 overexpression altered chromatin conformation during early preimplantation phases[42]. More recently, Oct4 has been shown to relax chromatin in 8-cell embryos [43]. These findings supported the conclusion in the present study that Oct4 is a functional chromatin remodeler around *Xist/Tsix* genomic loci. However, the mechanism by which Oct4 induces chromatin decondensation remains unknown. RNA-Seq analysis showed that the expression levels of major chromatin remodelling factor genes such as *Caf1*, *Brg1*, *Ring1b*, and *Ezh2* [44–46] were not dramatically altered by *Oct4* depletion (S2 Table).

One of the other possibilities for controlling chromatin remodeling is direct binding of Oct4 around *Xist*/*Tsix* regions. In ES cells, Oct4 could bind to XqD regions including *Xist/Tsix* loci [47] (S10 Fig). Moreover, recent study revealed that Nanog was necessary for an open heterochromatin organization in ES cells by direct binding to major satellite regions [48]. Thus, the direct bindings of Oct4 around *Xist/Tsix* loci might recruit transcriptional activators or evict transcriptional repressors.

### RNF12-mediated *Xist* activation

RNF12 is an essential factor for imprinted XCI [8]. The role of RNF12 as a dose-dependent *Xist* activator [7,49] is supported by the present study (Fig 7b). At late preimplantation embryos, as shown in previous studies using differentiating ES cells [20,21], *Rnf12* controls Xm-*Xist* expression by silencing *Tsix*, which was induced by Rex1 in XmXm embryos (Fig 7b). Thus, the primary role of *Rnf12* at late preimplantation phases is the silencing of *Rex1* leading to *Tsix* repression. However, the *Rnf12* expression levels of XmY and XmXp embryos markedly declined compared with those of XmXm embryos (Fig 4b). Therefore, under the physiological conditions of XmY and XmXp embryos, *Rnf12* double dosage never occurs and *Tsix* can be expressed from the Xm allele to induce chromatin alteration at *Xist* promoter regions.

In contrast, the role of RNF12 in Xp-*Xist* activation at early preimplantation phases remains a large question. Makhlouf et al. demonstrated that YY1 binds to *Xist* exon1 loci and can activate *Xist* in somatic cells [24]. These YY1 binding sites are CpG regions and DNA methylation inhibited this YY1 binding [24]. In support of the importance of YY1 binding sites for *Xist* activation, a DNA methylome study revealed that a part of the exon1 regions in the sperm genome were hypomethylated [50], implying YY1 binding in Xp-*Xist*. Furthermore, Gontan et al. showed the interaction of RNF12 with YY1 [20]. Therefore, the examination of the role of YY1 for Xp-*Xist* activation will aid in determining the mechanism of RNF12-mediated Xp-*Xist* activation.

## Materials and Methods

### Oocyte and sperm collection

Female B6D2F1 and male C57BL/J mice were purchased from CLEA and Sankyo Labo service (Japan) and oocytes and sperm were collected according to standard methods [4]. Repeat-A deletion mice were obtained from RIKEN BRC (B6.Cg-Xist<tm5Sado>). All animals were maintained and used in accordance with the Guidelines for the Care and Use of Laboratory Animals of the Japanese Association for Laboratory Animal Science and the National Research Institute for Child Health and Development (NRICHD) of Japan. All animal experiments were performed according to protocols approved by the Institutional Animal Care and Use Committee of the NRICHD (Permit Number: 05–006).

### Embryo manipulations

The production of parthenogenetic and androgenetic embryos was previously described [4]. In brief, oocytes were incubated in Ca-free M16 medium containing 8 mM SrCl_2_ and 5 μg/mL cytochalasin B (Sigma-Aldrich) for 5–6 hours. For production of haploid parthenogenetic embryos (hPE), the cytochalasin B was removed in the activation medium. All embryos were cultured in KSOM medium (EMD Millipore) in an atmosphere containing 5% CO_2_ at 37°C. In the TSA experiment, the embryos were cultured for 25 h in activation and culture media containing 50 nM TSA (Sigma-Aldrich). siRNAs were purchased from Life Technologies; siRNA sequences are described in S3 Table. siRNA injection into ovulated oocytes was conducted using a Piezo drive (Sutter Instrument Company). For expression or FISH experiments, the embryos were collected at 24–26 (2-cell), 48–50 (4-cell), 57–59 (8-cell), and 72–74 (morula) h after activation or insemination, respectively.

For nuclear transfer, HVJ-E (Ishihara Sangyo, Japan)-mediated fusion methods were used for all nuclear transfer experiments. Prior to nuclear transfer, zona pellucida was silted by a grass knife and the 1^st^ polar body was removed to prevent fusion with oocytes by HVJ-E. In male pronuclear transfer experiments, large pronucleus was selected and transferred into hPE. For the preparation of metaphase nuclei of hPE, hPE at the morula stage were incubated with M2 containing 1 μg/mL Nocodazole (Sigma-Aldrich) for 4–5 hours and used as donor cells. The reconstructed oocytes were subjected to intracytoplasmic sperm injection.

For embryo transfer, pseudopregnant ICR mice (Clea Japan) were used as embryo recipients. At E19.5, the embryos were recovered from the uterus.

### *In vitro* mRNA synthesis

The preparation of *in vitro* synthesized *Kdm4b* and *Egfp* mRNA was described previously [4]. For *Rnf12* mRNA synthesis, the full length coding sequence (CDS) was amplified by PCR using KOD-Plus-Neo DNA polymerase (Toyobo, Osaka, Japan) from 1-cell embryos. The amplified DNA was used as a template for the generation of PCR products with Poly-A tail and a T7 promoter and the products were subjected to *in vitro* transcription. The primers used for Rnf12 CDS amplification are shown in S3 Table.

### qPCR analysis of morula embryos

The qPCR analysis was conducted using TaqMan probes (Life Technologies) as described previously [4]. Total RNA from morula embryos (72 h after activation) was extracted using an RNeasy micro kit (Qiagen) according manufacturer instructions. *Gapdh* (Mm99999915_g1) was used as an internal control for normalization of target genes (*Oct4*: Mm00658129_gH, *Yy1*: Mm00456392_m1, and *Rex1*: Mm01194090_g1)

### Single cell qPCR analysis of preimplantation embryos

The zona pellucida was removed by treatment with acid Tyrode’s solution (Sigma) and single cells from each preimplantation stage were collected using a micromanipulator. Total RNA isolation and cDNA synthesis were performed using a Single Cell-to-CT^™^ qRT-PCR Kit (Thermo Fisher) with slight modifications. In brief, half volumes of all reagents were used in this study. The qPCR analysis using TaqMan probes (*Rnf12*: Mm00488044_m1 *Xist*: Mm01232884_m1) was conducted without a cDNA preamplification step. A total of 4 or more embryos were randomly selected from which to collect single cells used in the assay. The remaining cells at the 2-cell stage in fertilized embryos were subjected to DNA-FISH analysis as described below.

### Immunofluorescence

Embryos were fixed and permeabilised as previously described [13]. In brief, zona pellucida embryos were fixed with 2% PFA in PBS containing 0.1% PVA (Sigma) for 15 min at room temperature and then permeabilised with 0.25% Triton-X in PBS-PVA for 10 min at room temperature. After blocking with 1% BSA, the samples were incubated with the primary antibody RNF12 (1:500 diluted by blocking buffer, Abnova, H00051132-M01), H3K27me3 (1:200, Millipore, 07–449), or Oct4 (1:200, Santa Cruz Biotechnology, C-10), respectively. For H3K9me3 (1:500, Abcam, ab8898) and H3K9Ac (1:500, Abcam, ab12179) detection, fixation and permeabilisation treatments were simultaneously conducted and the primary antibodies were simultaneously incubated. The images were observed using a LSM510 laser scanning confocal microscope (Carl Zeiss). For quantification of the signal intensity, the same laser intensity was applied to each sample and the signals were calculated using U.S. National Institutes of Health (NIH) ImageJ software (http://rsb.info.nih.gov/ij/).

### eChIP-qPCR

Embryo-ChIP (eChIP) analysis for preimplantation embryos was based on previous reports. At least 15 XmXm morulae were used per assay. The primer/probe sequences used were described previously [13]. In addition, antibodies for H3K9me3 (Abcam, ab8898) and H3K27me3 (Millipore, 07–449) were used.

### RNA-FISH

The samples for RNA-FISH were prepared as previously described [4]. In brief, for *Xist* detection, the pXist12.9 plasmid containing the majority of the *Xist* cDNA was used (kindly gifted by T. Sado). For *Tsix* detection, the region (around 7 kb) of the *Tsix* locus from chr X: 103,448,873 to 103,455,853 was amplified by PCR and the products were subjected to nick translation (Abbott Laboratories). The region from chr X: 103,459,241 to 103,460,958 was amplified by PCR and cloned in the PUC118 vector (Takara), resulting in PCU118-Tsix1.7. The plasmid was also subjected to nick translation along with the PCR products of the 7 kb region. The FISH images were observed using a LSM510 laser scanning confocal microscope using C-.Apochromat 40x/1.2 W (Carl Zeiss).

### DNA-FISH

The DNA-FISH procedures were based on a previous study [13]. The fixed and permeabilised embryos were treated with RNaseA and then incubated with 0.2N HCl containing 0.05% tween-20 solution on ice for 10 min. The samples were incubated at 85°C for 10 min and then for overnight at 37°C. BAC DNA probes (RP23-311P7 and RP23-36C20) were prepared by nick translation. For evaluation of chromosome pairing, the probe derived from RP23-311P7 was used. Both probes were used for the chromatin condensation assay. For embryo sexing, the probes of X-chromosome (XqF4 regions) and Y-chromosome were purchased from Chromosome Science Labo (Sapporo, Japan). Distance measurements were based on previous reports [13]. Briefly, the signal centroid was calculated by NIH ImageJ software. Each nuclear radius used for distance normalization was calculated using the DAPI-stained area measurement. For image capture of all DNA FISH analyses, LSM510 laser scanning confocal microscopy using a Plan-Apochromat 100×/1.46 Oil DIC objective (Carl Zeiss) was used.

### RNA/DNA-FISH

Morula stage embryos were used for RNA/DNA-FISH analysis. The RNA-FISH procedure and image capture were carried out as in the above method and after image capture, the samples were washed with PBS and incubated with RNaseA for 1.5 h. After washing, the samples were treated with a solution including 0.01N HCl, 0.1% Tween20, and 100 μg/ml Pepsin (Sigma) for 7 min at 37°C. After washing, the samples were hybridized with probes at 85°C for 10 min and then overnight at 37°C. The image capture and distance calculations were performed as described above.

### Immunofluorescence combined with RNA/DNA-FISH

The IF-RNA/DNA-FISH procedures were based on a previous report [51]. In brief, the embryos were fixed with 2% PFA-PVA for 15 min at RT and then permeabilised with 0.25% Triton X-100 in PBS-PVA for 10 min. After washing with PBS-PVA, the samples were blocked in 1% BSA-PBS-PVA containing 1.3 U ml^−1^ RNaseOUT (Life Technologies) for 40 min. After washing, the embryos were incubated with primary antibodies (anti-RNF12, Abnova, diluted 1:200 in blocking buffer containing RNaseOUT) for 1 h. After incubation with the secondary antibody, the samples were subjected to RNA/DNA-FISH as described above except that the pepsin treatment in blastocysts was for 4 min.

### Transcriptome analysis

The Hiseq system (Illumina, Inc.) was used for RNA-sequencing. In brief, total RNA from each sample (30 pooled embryos) or single blastocysts were extracted using a Qiagen RNeasy Micro Kit (Qiagen), and the remaining DNA was degraded by DNase treatment. In blastocyst samples, a fraction of the total RNA was used for qPCR analysis to screen female samples. For Kdm4b/TSA-XmXp^Δ^ samples, we selected samples with high *Xist* expression. For construction of sequencing libraries, we used an Ovation Single Cell RNA-Seq System (NuGEN) according the manufacturer’s instruction. BAM format data yielded by Tohat 2.0.11 were subjected to successive analyses using AvadisNGS 1.6 (Agilent Technologies). The counts of raw reads allocated for each gene/transcript, which link to UCSC transcripts, were normalized using the TMM method (AvadisNGS 1.6). Normalized values were described as log2 values. For clustering analysis, the R function “hclust” (https://www.r-project.org/) was used to produce unsupervised clustering. The raw data was deposited in SRA (http://www.ncbi.nlm.nih.gov/sra) under accession I.D.: PRJNA312739 and PRJNA305455.

### Oct4 binding regions in ES cells

The published data of Oct4 ChIP-seq [52] (GSM566277) in ES cells was visualized via the UCSC genome browser (https://genome.ucsc.edu/) using custom tracks.

## Supporting Information

S1 Fig Effect of *Kdm4b* mRNA injection and TSA treatment on *Xist* expression state in XmXm embryos. (a) IF analysis of H3K9me3 and H3K9ac in Kdm4b/TSA-XmXm 2-cell embryos. For control embryos, *Egfp* mRNA was injected and cultured with DMSO (Egfp/DMSO). The same leaser intensity was applied to all samples. Blue, red, and green show DAPI, H3K9ac, and H3K9me3, respectively. (b) RNA-FISH analysis in Kdm4b/TSA-XmXm embryos at the 4-cell stage. n, the number of cells analysed. The P-values were calculated by the Fisher’s exact test. Scale bars show 20 μm.
(TIF)

S2 Fig *Xist* expression states Kdm4b-XmXp^Δ^ embryos. RNA/DNA-FISH analysis in Kdm4b overexpressing blastocysts. Representative images of XmY and XmXp^Δ^ embryos are shown. Circles represent individual embryos in lower graph. n, number of embryos analyzed. Scale bars, 20 μm.
(TIF)

S3 Fig *Xist*/*Tsix* expression states in Kdm4b/TSA-XmXp^Δ^ embryos. (a) Schematic view of RNA-FISH probes. *Xist/Tsix* and *Tsix* signals are shown in green and red, respectively. (b and c) RNA-FIHS analysis of *Xist*/*Tsix* in Kdm4b/TSA-XmY (b) and -XmXp^Δ^ (c). The sexing of embryos was determined by DNA-FISH (see methods). (d and e) qPCR analysis in individual blastocysts in XmY of WT, Egfp/DMSO, and Kdm4b/TSA treated embryos (d) and XmXp (WT), XmXp^Δ^ of control and Kdm4b/TSA treated embryos (e). The sexing of embryos was based on the presence of Eif2s3y mapped on the Y-chromosome. (f) Immunofluorescence analysis of H3K27me3 in Kdm4b/TSA treated embryos (Kdm4b/TSA-XmY or -XmXp^Δ^).
(TIF)

S4 Fig Differentially expressed genes compared with WT. Venn diagram shows differentially expressed genes (DEGs) in each group. Upregulated (a) and downregulated (b). The average expression levels of each group were used for analysis and > 3-fold genes compared with WT were identified as DEGs.
(TIF)

S5 Fig *Xist/Tsix* expression profiles in XmXp, XmY, and XmXm embryos during preimplantation phases. (a) RNA-FISH analysis in XmXp, XmY, and XmXm embryos during preimplantation stages. *Xist/Tsix* and *Tsix* signals are shown in green and red, respectively. Representative images (b). Quantification of FISH signal patterns. n, the number of cells analysed.
(TIF)

S6 Fig Examination of knockdown efficiency of Rnf12 and Rex1. (a) qPCR analysis of Rnf12KD-XmXm morulae. (b) Immunofluorescence analysis of RNF12 in Rnf12KD-XmXm morulae. Representative images were shown in picture and the graph showed signal intensities. The P-values were calculated by the Mann–Whitney U test. (c) qPCR analysis of Rex1KD-XmXm morulae. For qPCR analysis, pooled XmXm morulae were analyzed with two to three biological replicates. It was noted that we could not obtain antibody reacted to mouse REX1. The error bars show standard errors.
(TIF)

S7 Fig qPCR screening of pluripotency-related genes that potentially silence Xm-*Xist*. (a) The expression of *Xist* was examined in XmXm morula embryos treated with siRNA injection (*Oct4* or *Yy1*). Two to three independent experiments were conducted for each target gene. The error bars show standard errors. Expression levels of scramble controls were set to one. (b) IF analysis to examine the knockdown efficiency of OCT4 protein at the morula stage. n, the number of cells. The scale bars show 20 μm. The P-values were calculated using a student’s t-test. It was noted that we could not obtain an antibody reacted to mouse YY1. (c) RNA-FISH analysis in Oct4KD- and Yy1KD-XmXm morulae. The probes used for FISH detected *Xist*/*Tsix* signals.
(TIF)

S8 Fig Chromosome distributions of differentially expressed genes. The genes with over 2-fold changes compared with controls were identified as differentially expressed genes in Rnf12KD-XmXm (a) and Oct4KD-XmXm (b) embryos.
(TIF)

S9 Fig Effect of *Tet2* knockdown on Xm-*Xist* expression in XmXm embryos. (a) qPCR analysis of *Tet2* and *Xist* expression states. (b) Representative image of RNA-FISH using a *Xist/Tsix* detection probe. The graph showed quantification of *Xist* RNA-FISH results. The P-value was calculated by a Fisher’s exact test. n, the number of analysed cells.
(TIF)

S10 Fig Oct4 binding states in ES cells. ChIP-seq data of Oct4 in undifferentiated ES cells is shown using a UCSC custom track. The BAC probe regions used in this study are shown.
(TIF)

S1 Table RNA-seq data in Kdm4b/TSA-XmXp^Δ^, Egfp-XmXp^Δ^, and wild type female blastocysts. (XLSX)

S2 Table RNA-seq data in Oct4KD-XmXm, Rnf12KD-XmXm, scrable-XmXm morulae. (XLSX)

S3 Table Primer sequences. (XLSX)

## References

[1] Brockdorff N, Ashworth A, Kay GF, McCabe VM, Norris DP, et al (1992) The product of the mouse Xist gene is a 15 kb inactive X-specific transcript containing no conserved ORF and located in the nucleus. Cell 71: 515–526. 1423610 10.1016/0092-8674(92)90519-i

[2] Marahrens Y, Panning B, Dausman J, Strauss W, Jaenisch R (1997) Xist-deficient mice are defective in dosage compensation but not spermatogenesis. Genes Dev 11: 156–166. 9009199 10.1101/gad.11.2.156

[3] Penny GD, Kay GF, Sheardown SA, Rastan S, Brockdorff N (1996) Requirement for Xist in X chromosome inactivation. Nature 379: 131–137. 10.1038/379131a0 8538762

[4] Fukuda A, Tomikawa J, Miura T, Hata K, Nakabayashi K, et al (2014) The role of maternal-specific H3K9me3 modification in establishing imprinted X-chromosome inactivation and embryogenesis in mice. Nat Commun 5: 5464 10.1038/ncomms6464 25394724 PMC4243243

[5] Augui S, Nora EP, Heard E (2011) Regulation of X-chromosome inactivation by the X-inactivation centre. Nat Rev Genet 12: 429–442. 10.1038/nrg2987 21587299

[6] Barakat TS, Gunhanlar N, Pardo CG, Achame EM, Ghazvini M, et al (2011) RNF12 activates Xist and is essential for X chromosome inactivation. PLoS Genet 7: e1002001 10.1371/journal.pgen.1002001 21298085 PMC3029249

[7] Jonkers I, Barakat TS, Achame EM, Monkhorst K, Kenter A, et al (2009) RNF12 is an X-Encoded dose-dependent activator of X chromosome inactivation. Cell 139: 999–1011. 10.1016/j.cell.2009.10.034 19945382

[8] Shin J, Bossenz M, Chung Y, Ma H, Byron M, et al (2010) Maternal Rnf12/RLIM is required for imprinted X-chromosome inactivation in mice. Nature 467: 977–981. 10.1038/nature09457 20962847 PMC2967734

[9] Takagi N, Sasaki M (1975) Preferential inactivation of the paternally derived X chromosome in the extraembryonic membranes of the mouse. Nature 256: 640–642. 1152998 10.1038/256640a0

[10] Hoki Y, Kimura N, Kanbayashi M, Amakawa Y, Ohhata T, et al (2009) A proximal conserved repeat in the Xist gene is essential as a genomic element for X-inactivation in mouse. Development 136: 139–146. 10.1242/dev.026427 19036803

[11] Nesterova TB, Barton SC, Surani MA, Brockdorff N (2001) Loss of Xist imprinting in diploid parthenogenetic preimplantation embryos. Dev Biol 235: 343–350. 10.1006/dbio.2001.0295 11437441

[12] Fodor BD, Kubicek S, Yonezawa M, O'Sullivan RJ, Sengupta R, et al (2006) Jmjd2b antagonizes H3K9 trimethylation at pericentric heterochromatin in mammalian cells. Genes Dev 20: 1557–1562. 10.1101/gad.388206 16738407 PMC1482475

[13] Fukuda A, Mitani A, Miyashita T, Umezawa A, Akutsu H (2015) Chromatin condensation of Xist genomic loci during oogenesis in mice. Development 142: 4049–4055. 10.1242/dev.127308 26459223 PMC4712840

[14] Okamoto I, Tan S, Takagi N (2000) X-chromosome inactivation in XX androgenetic mouse embryos surviving implantation. Development 127: 4137–4145. 10976046 10.1242/dev.127.19.4137

[15] Aoki F, Worrad DM, Schultz RM (1997) Regulation of transcriptional activity during the first and second cell cycles in the preimplantation mouse embryo. Dev Biol 181: 296–307. 10.1006/dbio.1996.8466 9013938

[16] Adenot PG, Mercier Y, Renard JP, Thompson EM (1997) Differential H4 acetylation of paternal and maternal chromatin precedes DNA replication and differential transcriptional activity in pronuclei of 1-cell mouse embryos. Development 124: 4615–4625. 9409678 10.1242/dev.124.22.4615

[17] Sado T, Wang Z, Sasaki H, Li E (2001) Regulation of imprinted X-chromosome inactivation in mice by Tsix. Development 128: 1275–1286. 11262229 10.1242/dev.128.8.1275

[18] Lee JT, Davidow LS, Warshawsky D (1999) Tsix, a gene antisense to Xist at the X-inactivation centre. Nat Genet 21: 400–404. 10.1038/7734 10192391

[19] Plath K, Fang J, Mlynarczyk-Evans SK, Cao R, Worringer KA, et al (2003) Role of histone H3 lysine 27 methylation in X inactivation. Science 300: 131–135. 10.1126/science.1084274 12649488

[20] Gontan C, Achame EM, Demmers J, Barakat TS, Rentmeester E, et al (2012) RNF12 initiates X-chromosome inactivation by targeting REX1 for degradation. Nature 485: 386–390. 10.1038/nature11070 22596162

[21] Navarro P, Oldfield A, Legoupi J, Festuccia N, Dubois A, et al (2010) Molecular coupling of Tsix regulation and pluripotency. Nature 468: 457–460. 10.1038/nature09496 21085182

[22] Hamatani T, Carter MG, Sharov AA, Ko MS (2004) Dynamics of global gene expression changes during mouse preimplantation development. Dev Cell 6: 117–131. 14723852 10.1016/s1534-5807(03)00373-3

[23] Navarro P, Chambers I, Karwacki-Neisius V, Chureau C, Morey C, et al (2008) Molecular coupling of Xist regulation and pluripotency. Science 321: 1693–1695. 10.1126/science.1160952 18802003

[24] Makhlouf M, Ouimette JF, Oldfield A, Navarro P, Neuillet D, et al (2014) A prominent and conserved role for YY1 in Xist transcriptional activation. Nat Commun 5: 4878 10.1038/ncomms5878 25209548 PMC4172967

[25] Donohoe ME, Silva SS, Pinter SF, Xu N, Lee JT (2009) The pluripotency factor Oct4 interacts with Ctcf and also controls X-chromosome pairing and counting. Nature 460: 128–132. 10.1038/nature08098 19536159 PMC3057664

[26] Robinson MD, Oshlack A (2010) A scaling normalization method for differential expression analysis of RNA-seq data. Genome Biol 11: R25 10.1186/gb-2010-11-3-r25 20196867 PMC2864565

[27] Ito S, D'Alessio AC, Taranova OV, Hong K, Sowers LC, et al (2010) Role of Tet proteins in 5mC to 5hmC conversion, ES-cell self-renewal and inner cell mass specification. Nature 466: 1129–1133. 10.1038/nature09303 20639862 PMC3491567

[28] Chiba H, Hirasawa R, Kaneda M, Amakawa Y, Li E, et al (2008) De novo DNA methylation independent establishment of maternal imprint on X chromosome in mouse oocytes. Genesis 46: 768–774. 10.1002/dvg.20438 18932249

[29] Sun S, Del Rosario BC, Szanto A, Ogawa Y, Jeon Y, et al (2013) Jpx RNA activates Xist by evicting CTCF. Cell 153: 1537–1551. 10.1016/j.cell.2013.05.028 23791181 PMC3777401

[30] Chureau C, Chantalat S, Romito A, Galvani A, Duret L, et al (2011) Ftx is a non-coding RNA which affects Xist expression and chromatin structure within the X-inactivation center region. Hum Mol Genet 20: 705–718. 10.1093/hmg/ddq516 21118898

[31] Sado T, Hoki Y, Sasaki H (2005) Tsix silences Xist through modification of chromatin structure. Dev Cell 9: 159–165. 10.1016/j.devcel.2005.05.015 15992549

[32] Navarro P, Pichard S, Ciaudo C, Avner P, Rougeulle C (2005) Tsix transcription across the Xist gene alters chromatin conformation without affecting Xist transcription: implications for X-chromosome inactivation. Genes Dev 19: 1474–1484. 10.1101/gad.341105 15964997 PMC1151664

[33] Okamoto I, Patrat C, Thepot D, Peynot N, Fauque P, et al (2011) Eutherian mammals use diverse strategies to initiate X-chromosome inactivation during development. Nature 472: 370–374. 10.1038/nature09872 21471966

[34] Sado T, Sakaguchi T (2013) Species-specific differences in X chromosome inactivation in mammals. Reproduction 146: R131–139. 10.1530/REP-13-0173 23847260

[35] Tada T, Obata Y, Tada M, Goto Y, Nakatsuji N, et al (2000) Imprint switching for non-random X-chromosome inactivation during mouse oocyte growth. Development 127: 3101–3105. 10862747 10.1242/dev.127.14.3101

[36] Santos F, Peters AH, Otte AP, Reik W, Dean W (2005) Dynamic chromatin modifications characterise the first cell cycle in mouse embryos. Dev Biol 280: 225–236. 10.1016/j.ydbio.2005.01.025 15766761

[37] Puschendorf M, Terranova R, Boutsma E, Mao X, Isono K, et al (2008) PRC1 and Suv39h specify parental asymmetry at constitutive heterochromatin in early mouse embryos. Nat Genet 40: 411–420. 10.1038/ng.99 18311137

[38] Nakamura T, Liu YJ, Nakashima H, Umehara H, Inoue K, et al (2012) PGC7 binds histone H3K9me2 to protect against conversion of 5mC to 5hmC in early embryos. Nature 486: 415–419. 10.1038/nature11093 22722204

[39] Ma P, Pan H, Montgomery RL, Olson EN, Schultz RM (2012) Compensatory functions of histone deacetylase 1 (HDAC1) and HDAC2 regulate transcription and apoptosis during mouse oocyte development. Proc Natl Acad Sci U S A 109: E481–489. 10.1073/pnas.1118403109 22223663 PMC3286984

[40] Beuchat A, Thevenaz P, Unser M, Ebner T, Senn A, et al (2008) Quantitative morphometrical characterization of human pronuclear zygotes. Hum Reprod 23: 1983–1992. 10.1093/humrep/den206 18540007

[41] Wu G, Han D, Gong Y, Sebastiano V, Gentile L, et al (2013) Establishment of totipotency does not depend on Oct4A. Nat Cell Biol 15: 1089–1097. 10.1038/ncb2816 23934214 PMC3845671

[42] Fukuda A, Mitani A, Miyashita T, Kobayashi H, Umezawa A, et al (2016) Spatiotemporal dynamics of Oct4 protein localization during preimplantation development in mice. Reproduction. 10.1530/REP-16-0277 PMC5064760 27495230

[43] Lu F, Liu Y, Inoue A, Suzuki T, Zhao K, et al (2016) Establishing Chromatin Regulatory Landscape during Mouse Preimplantation Development. Cell 165: 1375–1388. 10.1016/j.cell.2016.05.050 27259149 PMC6625655

[44] Terranova R, Yokobayashi S, Stadler MB, Otte AP, van Lohuizen M, et al (2008) Polycomb group proteins Ezh2 and Rnf2 direct genomic contraction and imprinted repression in early mouse embryos. Dev Cell 15: 668–679. 10.1016/j.devcel.2008.08.015 18848501

[45] Cheloufi S, Elling U, Hopfgartner B, Jung YL, Murn J, et al (2015) The histone chaperone CAF-1 safeguards somatic cell identity. Nature 528: 218–224. 10.1038/nature15749 26659182 PMC4866648

[46] de Dieuleveult M, Yen K, Hmitou I, Depaux A, Boussouar F, et al (2016) Genome-wide nucleosome specificity and function of chromatin remodellers in ES cells. Nature 530: 113–116. 10.1038/nature16505 26814966 PMC4871117

[47] Chen X, Xu H, Yuan P, Fang F, Huss M, et al (2008) Integration of external signaling pathways with the core transcriptional network in embryonic stem cells. Cell 133: 1106–1117. 10.1016/j.cell.2008.04.043 18555785

[48] Novo CL, Tang C, Ahmed K, Djuric U, Fussner E, et al (2016) The pluripotency factor Nanog regulates pericentromeric heterochromatin organization in mouse embryonic stem cells. Genes Dev 30: 1101–1115. 10.1101/gad.275685.115 27125671 PMC4863740

[49] Barakat TS, Loos F, van Staveren S, Myronova E, Ghazvini M, et al (2014) The trans-activator RNF12 and cis-acting elements effectuate X chromosome inactivation independent of X-pairing. Mol Cell 53: 965–978. 10.1016/j.molcel.2014.02.006 24613346

[50] Kobayashi H, Sakurai T, Imai M, Takahashi N, Fukuda A, et al (2012) Contribution of intragenic DNA methylation in mouse gametic DNA methylomes to establish oocyte-specific heritable marks. PLoS Genet 8: e1002440 10.1371/journal.pgen.1002440 22242016 PMC3252278

[51] Namekawa SH, Lee JT (2011) Detection of nascent RNA, single-copy DNA and protein localization by immunoFISH in mouse germ cells and preimplantation embryos. Nat Protoc 6: 270–284. 10.1038/nprot.2010.195 21372809 PMC4335666

[52] Ang YS, Tsai SY, Lee DF, Monk J, Su J, et al (2011) Wdr5 mediates self-renewal and reprogramming via the embryonic stem cell core transcriptional network. Cell 145: 183–197. 10.1016/j.cell.2011.03.003 21477851 PMC3097468

