# Supplementary figures and images for: Maintenance of Xist Imprinting Depends on Chromatin Condensation State and Rnf12 Dosage in Mice
Source: PLoS Genet. 2016 Oct 27;12(10):e1006375. doi: 10.1371/journal.pgen.1006375 (PMC5082930; doi:10.1371/journal.pgen.1006375)

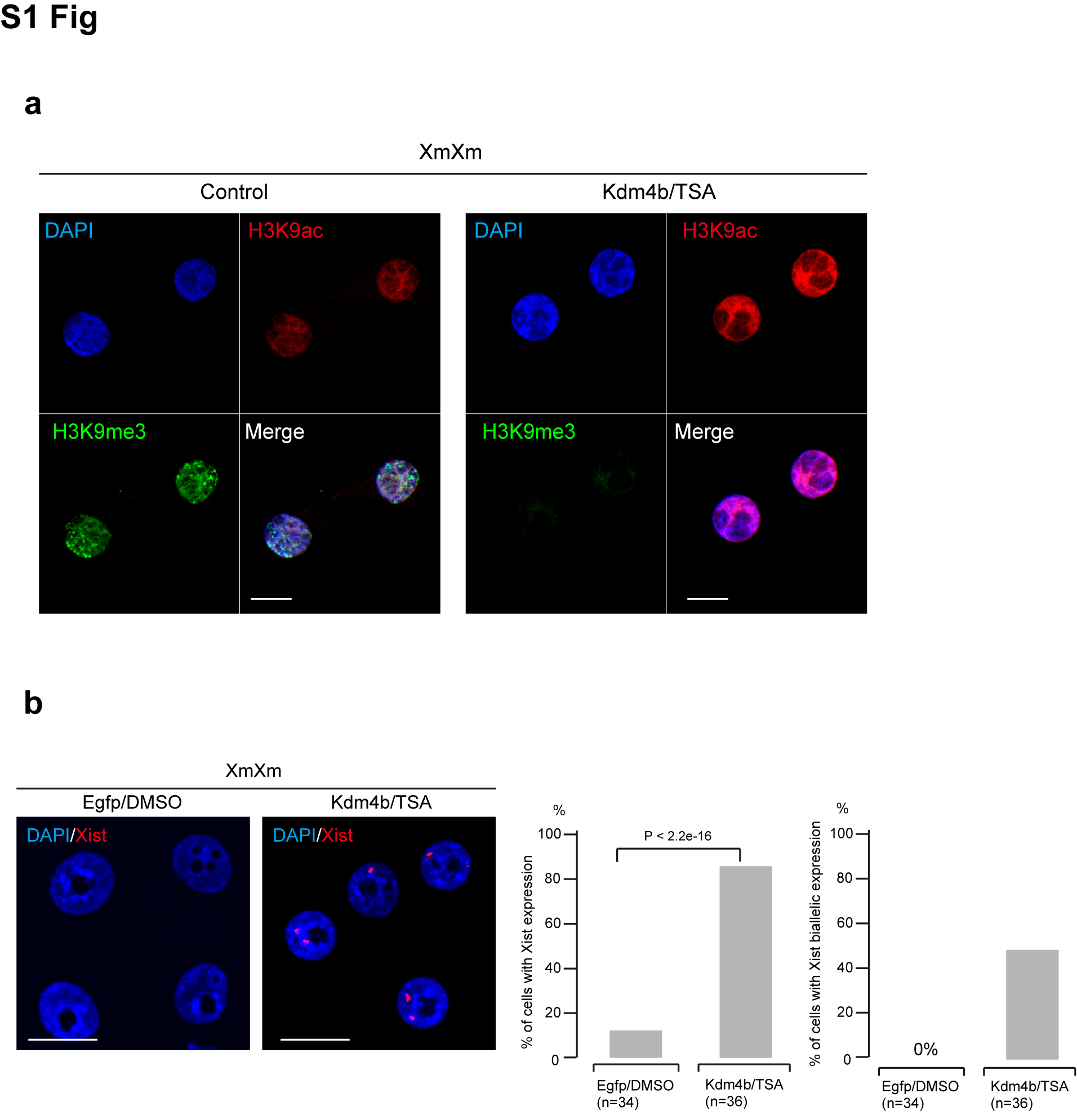

Supplement: S1 Fig — (a) IF analysis of H3K9me3 and H3K9ac in Kdm4b/TSA-XmXm 2-cell embryos. For control embryos, Egfp mRNA was injected and cultured with DMSO (Egfp/DMSO). The same leaser intensity was applied to all samples. Blue, red, and green show DAPI, H3K9ac, and H3K9me3, respectively. (b) RNA-FISH analysis in Kdm4b/TSA-XmXm embryos at the 4-cell stage. n, the number of cells analysed. The P-values were calculated by the Fisher’s exact test. Scale bars show 20 μm. (TIF) [file pgen.1006375.s001.tif]

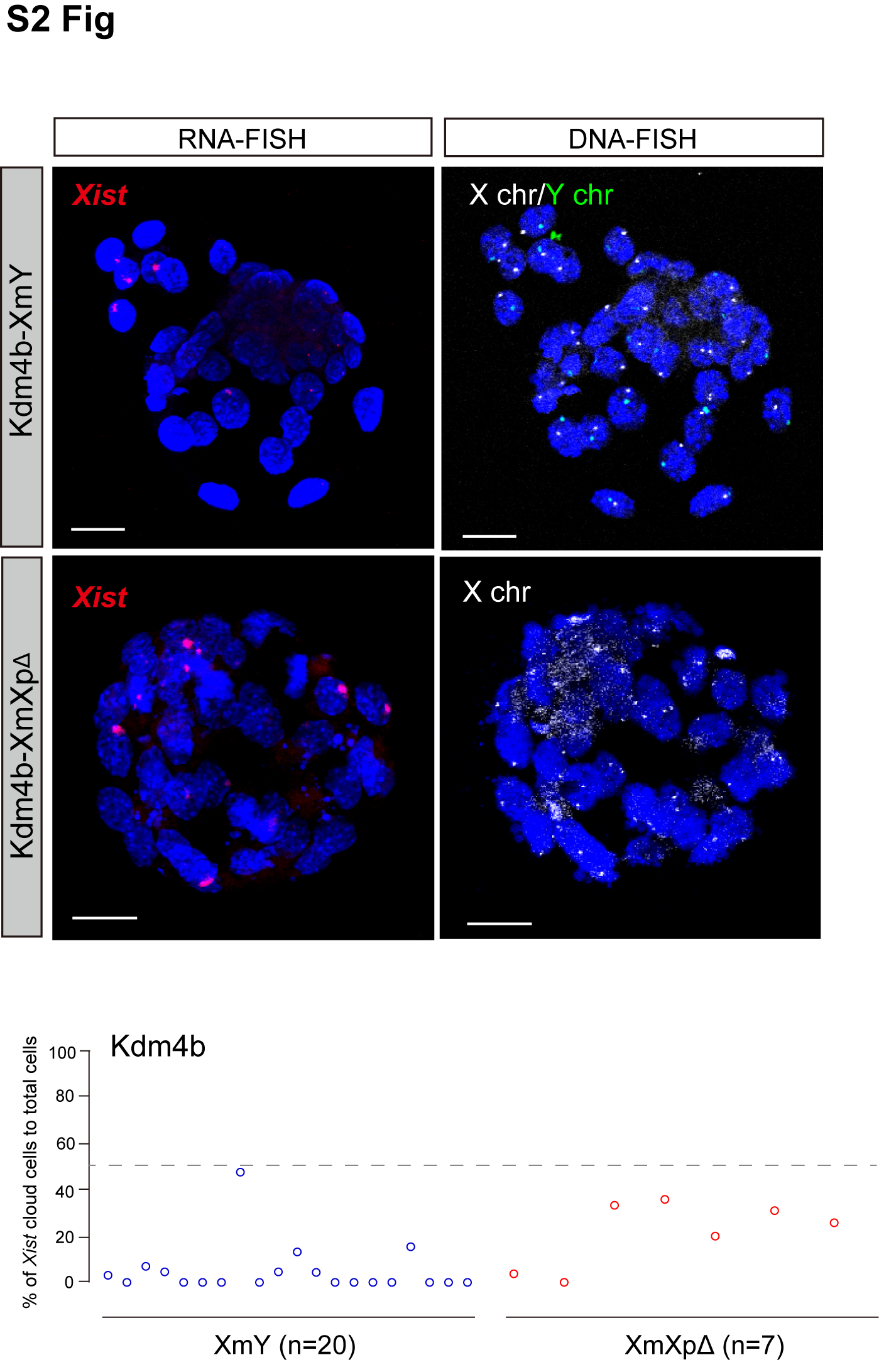

Supplement: S2 Fig — RNA/DNA-FISH analysis in Kdm4b overexpressing blastocysts. Representative images of XmY and XmXpΔ embryos are shown. Circles represent individual embryos in lower graph. n, number of embryos analyzed. Scale bars, 20 μm. (TIF) [file pgen.1006375.s002.tif]

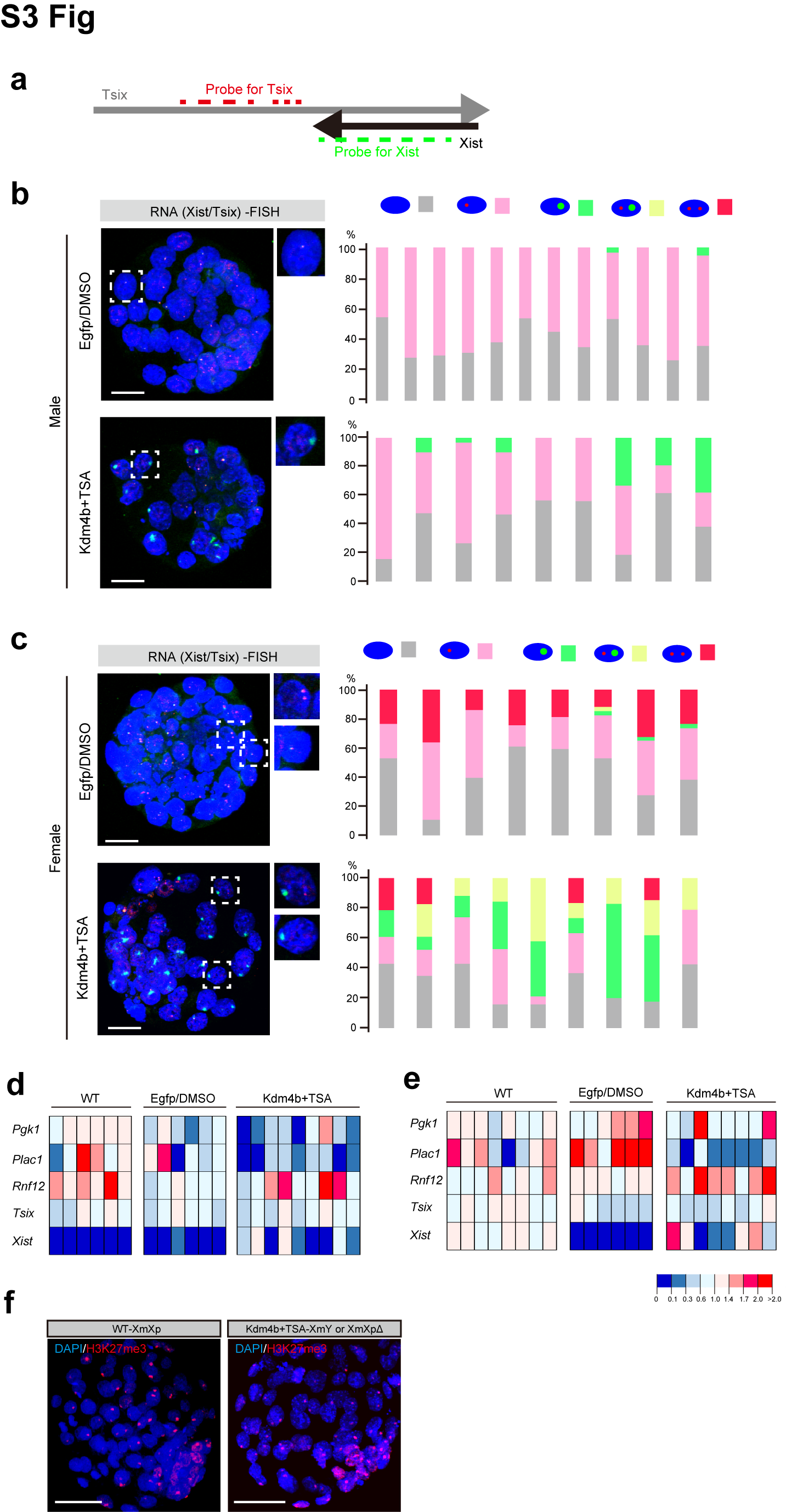

Supplement: S3 Fig — (a) Schematic view of RNA-FISH probes. Xist/Tsix and Tsix signals are shown in green and red, respectively. (b and c) RNA-FIHS analysis of Xist/Tsix in Kdm4b/TSA-XmY (b) and -XmXpΔ (c). The sexing of embryos was determined by DNA-FISH (see methods). (d and e) qPCR analysis in individual blastocysts in XmY of WT, Egfp/DMSO, and Kdm4b/TSA treated embryos (d) and XmXp (WT), XmXpΔ of control and Kdm4b/TSA treated embryos (e). The sexing of embryos was based on the presence of Eif2s3y mapped on the Y-chromosome. (f) Immunofluorescence analysis of H3K27me3 in Kdm4b/TSA treated embryos (Kdm4b/TSA-XmY or -XmXpΔ). (TIF) [file pgen.1006375.s003.tif]

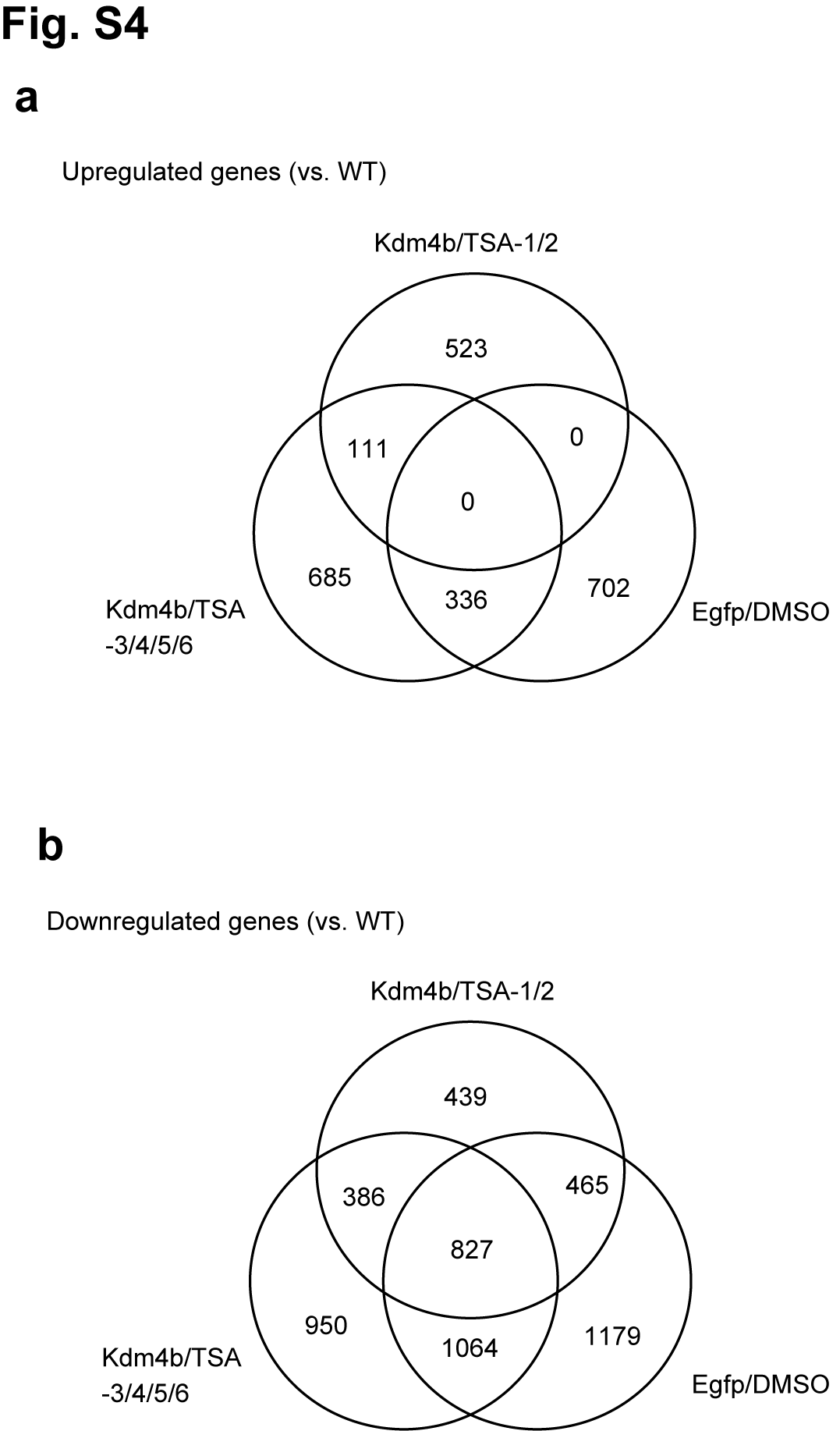

Supplement: S4 Fig — Venn diagram shows differentially expressed genes (DEGs) in each group. Upregulated (a) and downregulated (b). The average expression levels of each group were used for analysis and > 3-fold genes compared with WT were identified as DEGs. (TIF) [file pgen.1006375.s004.tif]

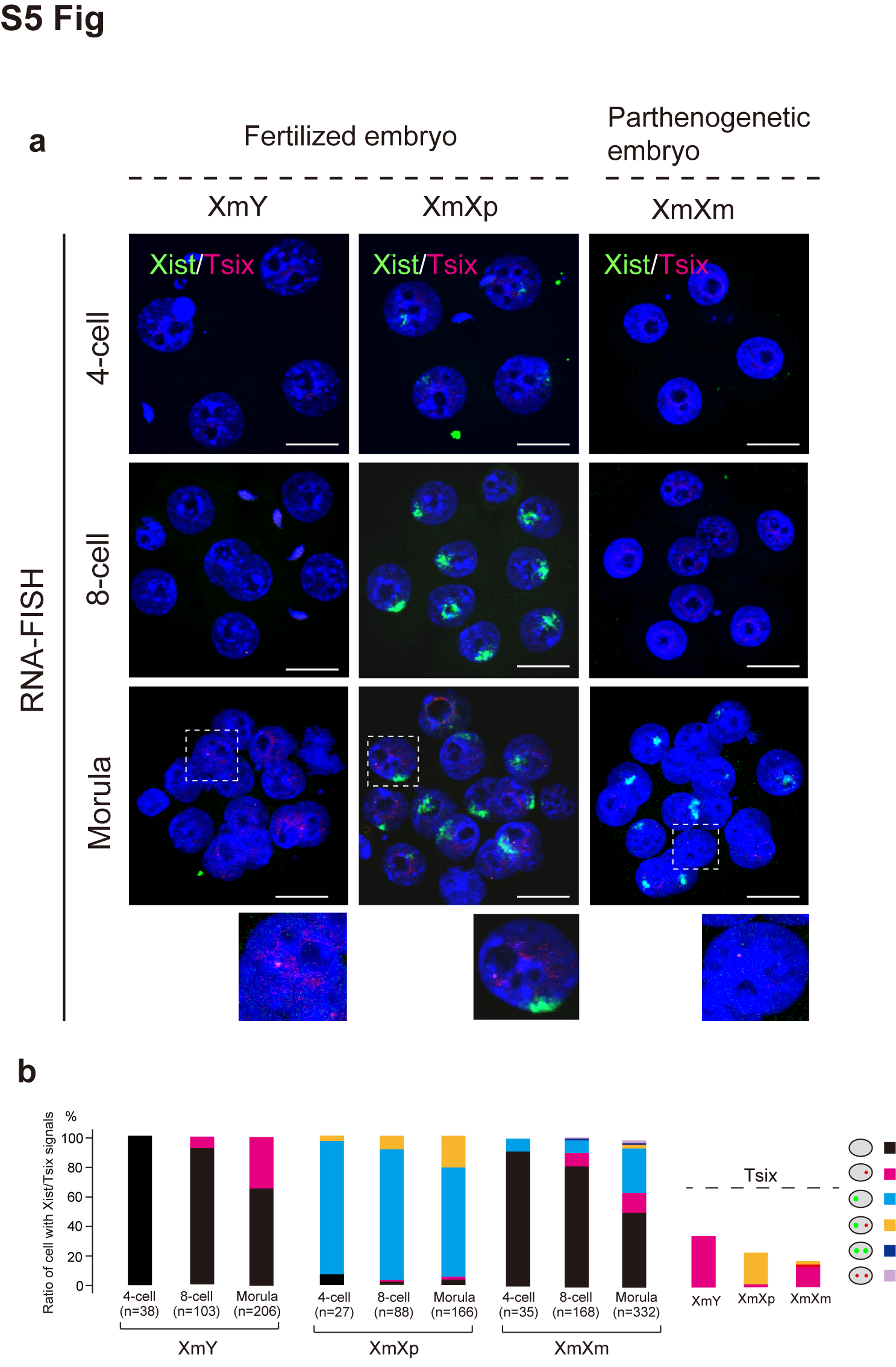

Supplement: S5 Fig — (a) RNA-FISH analysis in XmXp, XmY, and XmXm embryos during preimplantation stages. Xist/Tsix and Tsix signals are shown in green and red, respectively. Representative images (b). Quantification of FISH signal patterns. n, the number of cells analysed. (TIF) [file pgen.1006375.s005.tif]

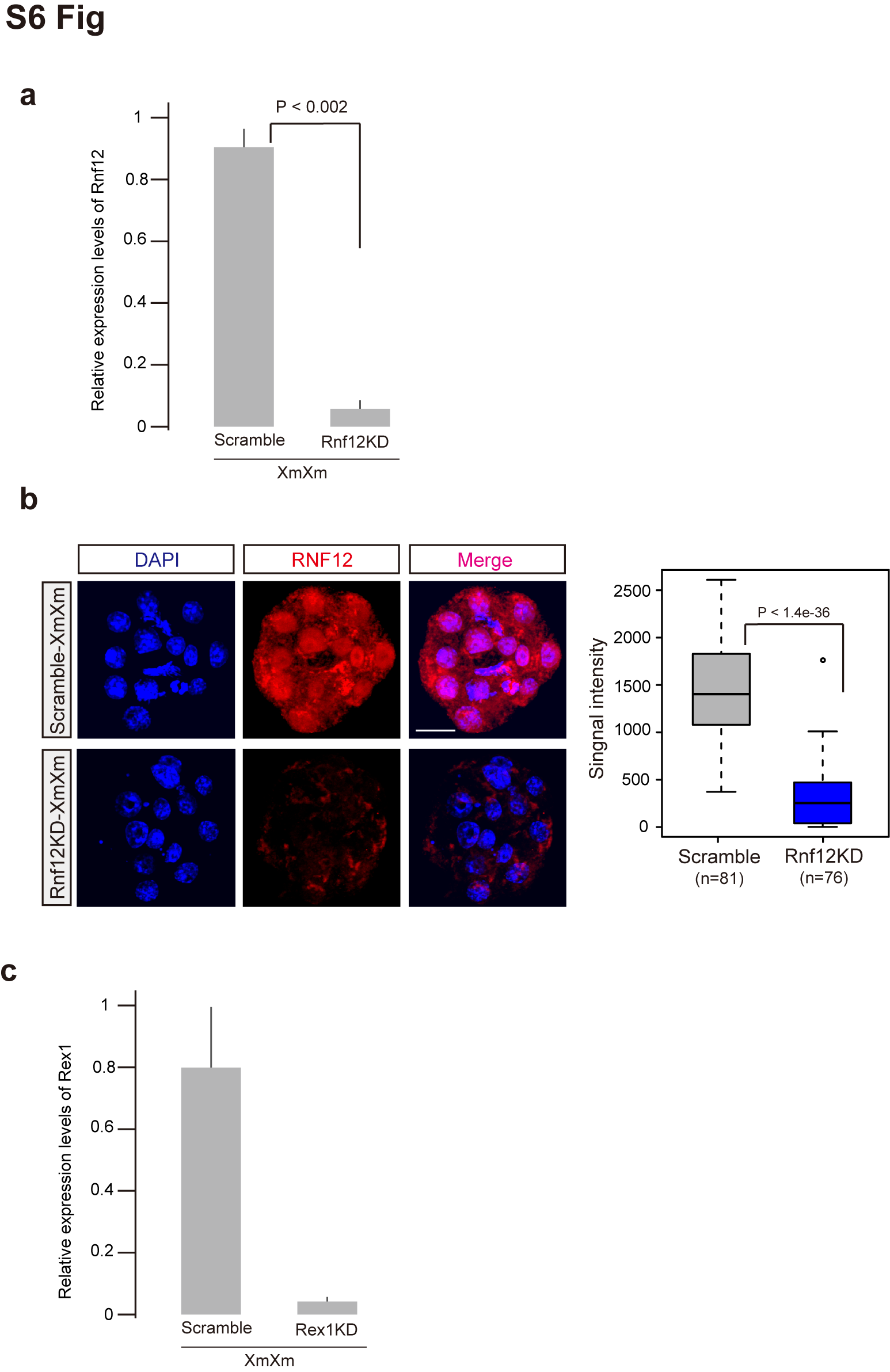

Supplement: S6 Fig — (a) qPCR analysis of Rnf12KD-XmXm morulae. (b) Immunofluorescence analysis of RNF12 in Rnf12KD-XmXm morulae. Representative images were shown in picture and the graph showed signal intensities. The P-values were calculated by the Mann–Whitney U test. (c) qPCR analysis of Rex1KD-XmXm morulae. For qPCR analysis, pooled XmXm morulae were analyzed with two to three biological replicates. It was noted that we could not obtain antibody reacted to mouse REX1. The error bars show standard errors. (TIF) [file pgen.1006375.s006.tif]

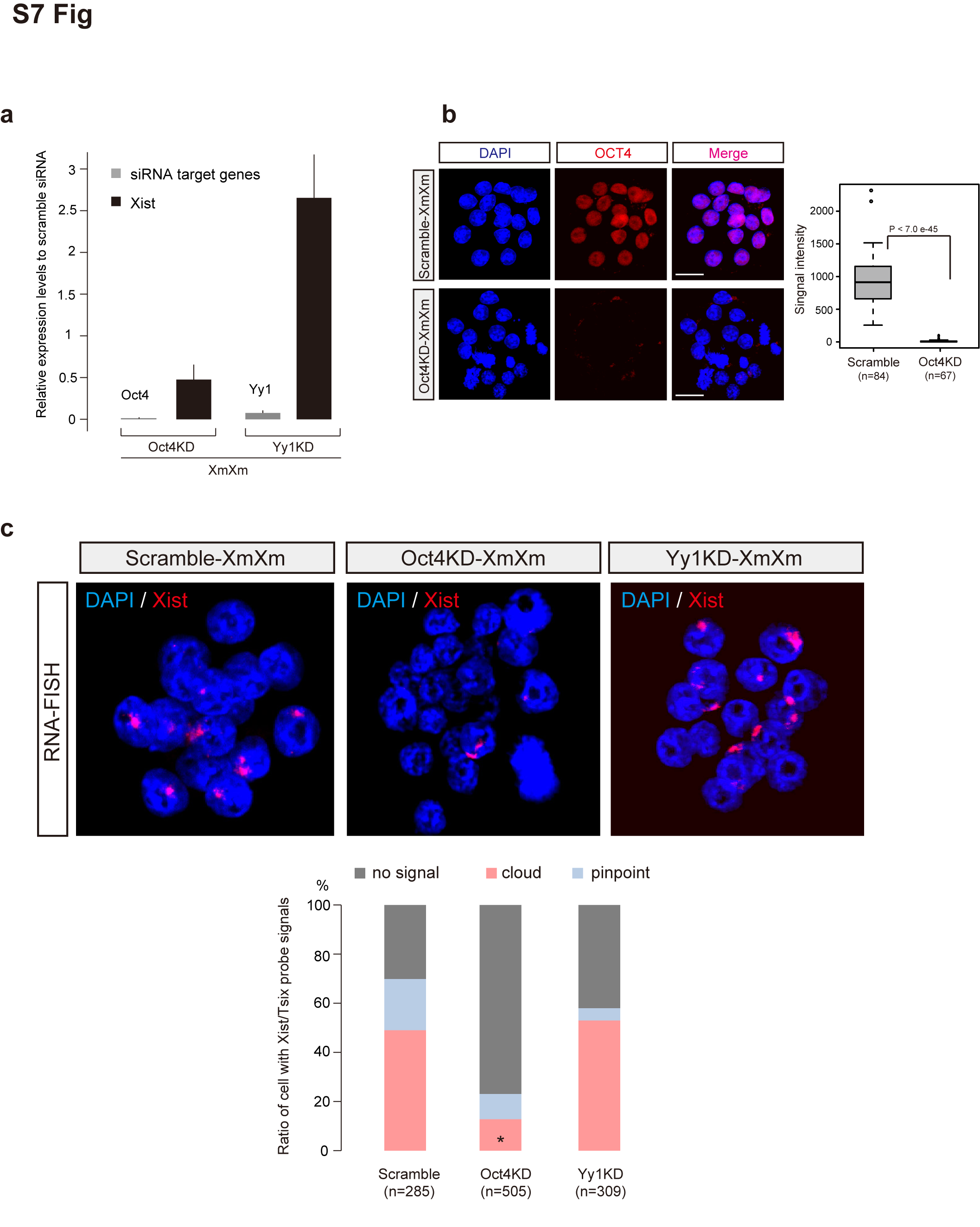

Supplement: S7 Fig — (a) The expression of Xist was examined in XmXm morula embryos treated with siRNA injection (Oct4 or Yy1). Two to three independent experiments were conducted for each target gene. The error bars show standard errors. Expression levels of scramble controls were set to one. (b) IF analysis to examine the knockdown efficiency of OCT4 protein at the morula stage. n, the number of cells. The scale bars show 20 μm. The P-values were calculated using a student’s t-test. It was noted that we could not obtain an antibody reacted to mouse YY1. (c) RNA-FISH analysis in Oct4KD- and Yy1KD-XmXm morulae. The probes used for FISH detected Xist/Tsix signals. (TIF) [file pgen.1006375.s007.tif]

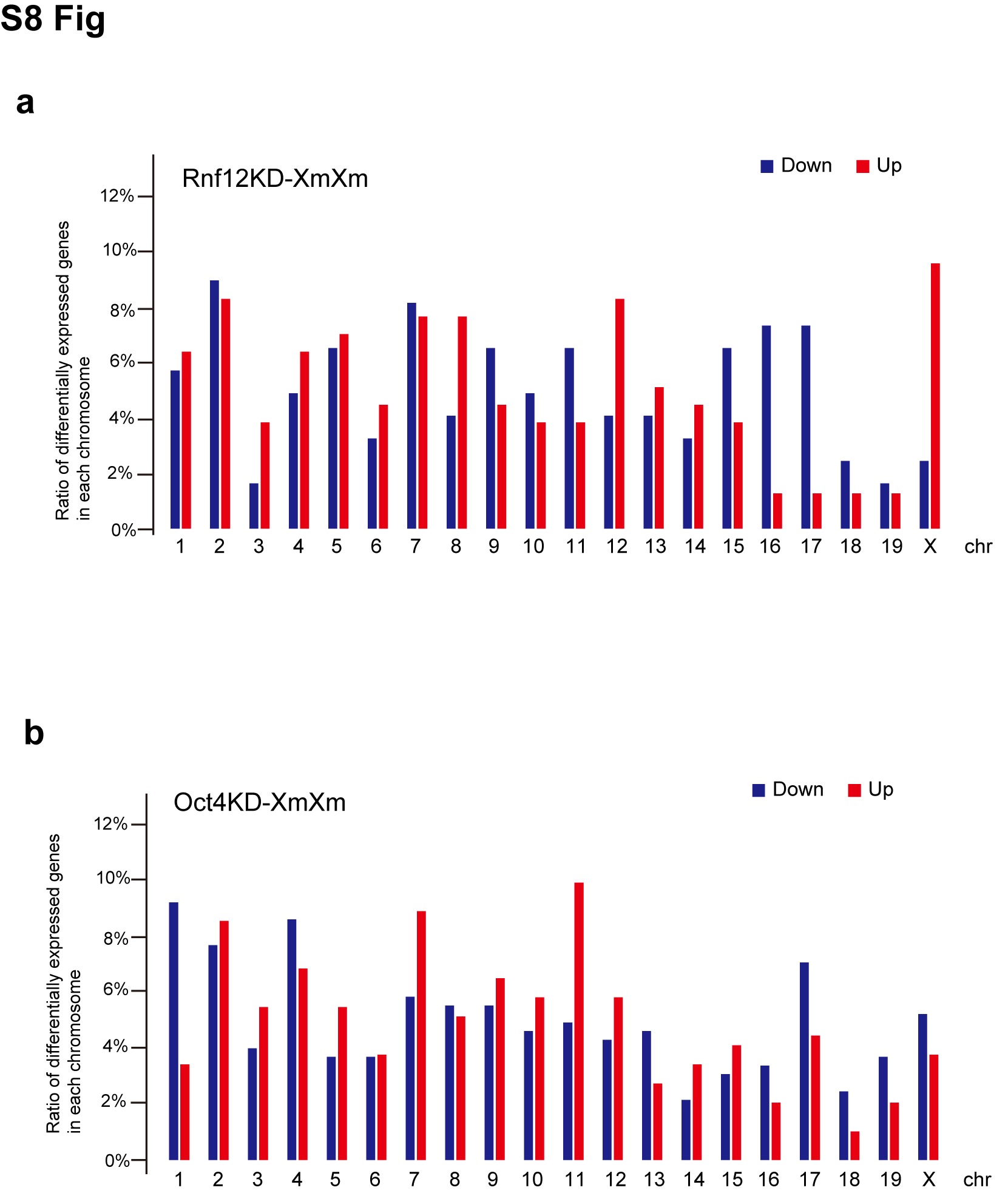

Supplement: S8 Fig — The genes with over 2-fold changes compared with controls were identified as differentially expressed genes in Rnf12KD-XmXm (a) and Oct4KD-XmXm (b) embryos. (TIF) [file pgen.1006375.s008.tif]

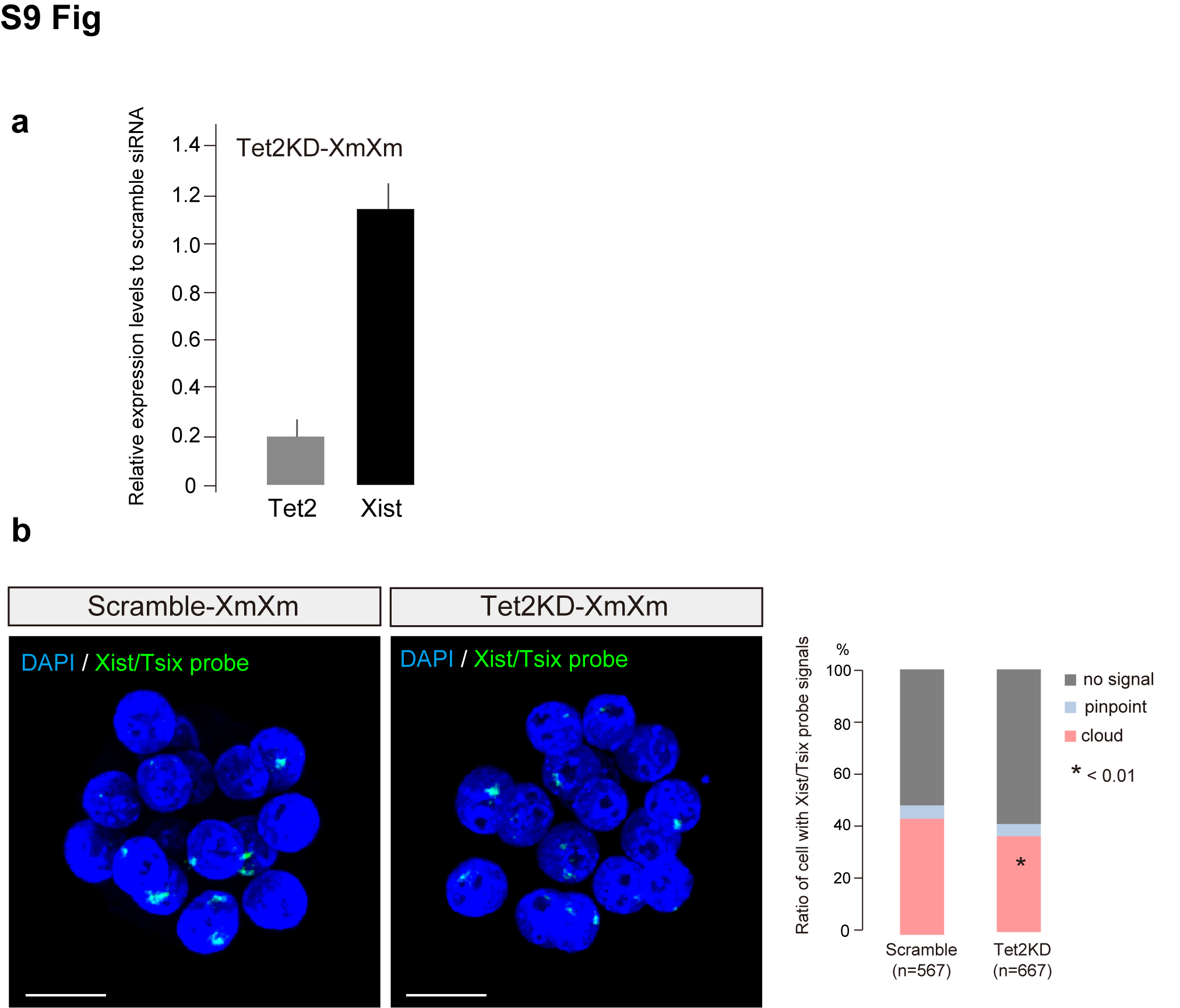

Supplement: S9 Fig — (a) qPCR analysis of Tet2 and Xist expression states. (b) Representative image of RNA-FISH using a Xist/Tsix detection probe. The graph showed quantification of Xist RNA-FISH results. The P-value was calculated by a Fisher’s exact test. n, the number of analysed cells. (TIF) [file pgen.1006375.s009.tif]

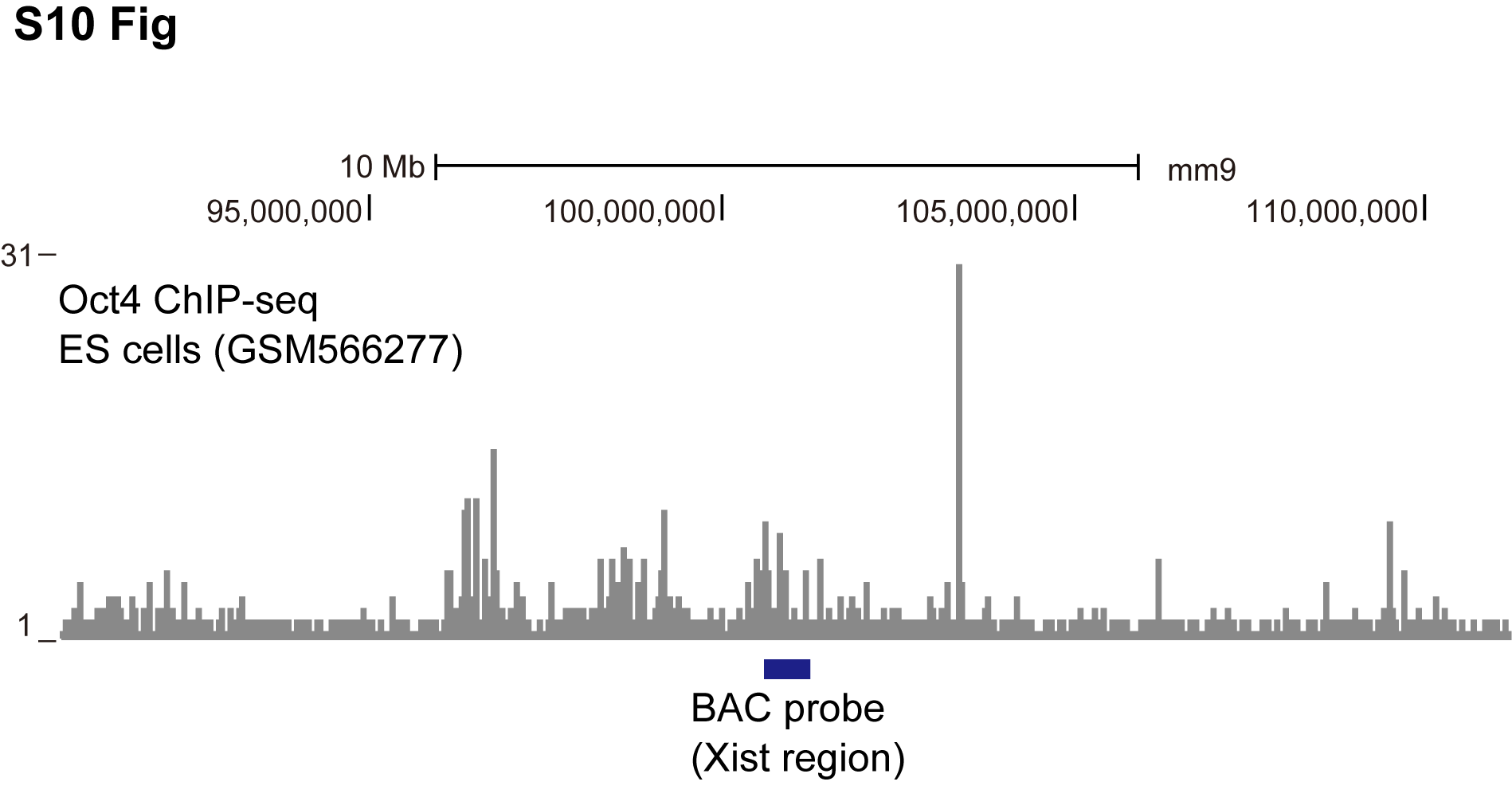

Supplement: S10 Fig — ChIP-seq data of Oct4 in undifferentiated ES cells is shown using a UCSC custom track. The BAC probe regions used in this study are shown. (TIF) [file pgen.1006375.s010.tif]
